# Intestinal Activation of LXRα Counteracts Metabolic-Associated Steatohepatitis Features in Mice

**DOI:** 10.3390/nu17081349

**Published:** 2025-04-15

**Authors:** Gessica Lioci, Fabio Gurrado, Nadia Panera, Marzia Bianchi, Cristiano De Stefanis, Valentina D’Oria, Nicolò Cicolani, Silvano Junior Santini, Laura Schiadà, Anna Alisi, Gianluca Svegliati-Baroni

**Affiliations:** 1 Liver Injury and Transplant Unit, Polytechnic University of Marche, 60121 Ancona, Italy; gessicalioci@gmail.com (G.L.); gurrado.fabio@gmail.com (F.G.); laura.schiada@ospedaliriuniti.marche.it (L.S.); gsvegliati@gmail.com (G.S.-B.); 2 Research Unit of Genetics of Complex Phenotypes, Bambino Gesù Children’s Hospital, IRCCS, 00165 Rome, Italy; nadia.panera@opbg.net (N.P.); marzia.bianchi@opbg.net (M.B.); 3 Core Facilities, Bambino Gesù Children’s Hospital, IRCCS, 00146 Rome, Italy; cristiano.destefanis@opbg.net (C.D.S.); valentina.doria@opbg.net (V.D.); nicolo.cicolani@opbg.net (N.C.); 4 Department of Life, Health and Environmental Sciences-MESVA, School of Emergency-Urgency Medicine, University of L’Aquila, 67100 L’Aquila, Italy; silvanojunior.santini@univaq.it

**Keywords:** inflammation, intestine, MASLD, MASH, liver fibrosis, liver X receptor, HDL, SRB1

## Abstract

**Background/Objectives**: Metabolic dysfunction-associated steatotic liver disease (MASLD) is a growing global health problem and the discovery of drugs is challenging. In this study, we aimed to investigate the effects of intestinal activation of the liver X receptor (LXR)α on MASH. **Methods**: An intestinal-specific LXRα activation model in mice was established and subjected to MASH development by combining a Western diet and carbon tetrachloride. Lipid metabolism, reverse cholesterol transport (RCT), steatosis, inflammation, and fibrosis were evaluated. In vitro models of steatosis and fibrosis were used to explore the role of scavenger receptor class B type 1 (SRB1). **Results**: We found that the intestinal activation of LXRα improved several MASLD features, including levels of triglycerides, RCT, steatosis, systemic and hepatic inflammatory profiles, and liver fibrosis. These effects were associated with increased high-density lipoprotein (HDL) levels and hepatic SRB1 expression. In vitro depletion of SRB1 hampered the beneficial effects of HDL on steatosis and fibrogenesis in liver cells by altering the activation of both peroxisome proliferator-activated receptors γ and small mothers against decapentaplegic homolog protein (SMAD)2/3 proteins. **Conclusions**: Our findings showed that the intestinal activation of LXRα and a parallel induction of hepatic SRB1 are protective against inflammation, steatosis, and advanced liver fibrosis in MASLD.

## 1. Introduction

Non-alcoholic fatty liver disease (NAFLD) is a global public health problem representing the most common form of chronic liver injury worldwide [1,2]. NAFLD, recently renamed metabolic dysfunction-associated steatotic liver disease (MASLD), is an umbrella of entities covering a spectrum of liver and metabolic disorders. MASLD exhibits heterogeneous pathophysiology with a progressive phenotype ranging from metabolic dysfunction-associated fatty liver (MAFL) to metabolic dysfunction-associated steatohepatitis (MASH), defined by histological evidence of lobular inflammation, cytologic ballooning, and some degree of fibrosis leading to hepatic cirrhosis and/or hepatocellular carcinoma [3,4]. At present, there are no therapies available for MASH.

The onset and progression of MASLD may be influenced by nutritional overload, genetic background, microbiota composition, and several epigenetic factors [5], ultimately leading to an excessive accumulation of fat in the liver [6]. In this multifactorial model, lipotoxicity is a primary mechanism that promotes the progression of MASH and fibrosis [7,8]. Free cholesterol accumulation in hepatocytes is a key driver of lipotoxicity [9,10]. In this context, liver X receptors (LXRα and LXRβ) may regulate signaling pathways of cholesterol efflux to promote cholesterol elimination from the liver [11,12]. In this regard, we demonstrated that, in a transgenic mice model, the intestinal activation of LXRα ameliorated the hepatic response during chronic liver injury caused by carbon tetrachloride (CCl4) [13].

Here, we analyzed the protective effects of intestinal LXRα activation on MASH features in a model combining CCl4 administration and Western diet (WD) feeding in mice. We found that specific intestinal activation of LXRα induced an increase in circulating HDL coupled with an increased expression of hepatic SRB1, which mediates the selective uptake of lipoprotein (HDL)-derived cholesteryl ester (CE), thus protecting against exacerbation of MASH. Moreover, we suggested that the beneficial effects of HDL were mediated by SRB1 action on peroxisome proliferator-activated receptor γ (PPARγ) and small mothers against decapentaplegic homolog protein 2/3 (SMAD2/3) proteins, which are involved in hepatic steatosis and fibrosis, respectively.

## 2. Materials and Methods

### 2.1. Animals

Parental mice with constitutive activation of LXRα in the intestine (iVP16LXRα) and their specific controls (iVP16) were kindly provided by Professor Antonio Moschetta (Department of Interdisciplinary Medicine, University of Bari “Aldo Moro”, Italy). The intestinal-specific VP16LXRα transgenic model was generated by injecting the pSKVillin-VP16LXRα plasmid digested with HpaI into the pronuclei of the fertilized eggs of FVB/N mice, as reported by Lo Sasso et al. [14].

Eight-week-old male iVP16 (wild-type, WT) mice (*n* = 12) and iVP16LXRα (iLXRα) mice (*n* = 12) were divided into the following two groups: the control group received a normal diet (ND) and normal tap water in addition to intraperitoneal injection of corn oil, once a week for 12 weeks; the htreated group received WD (TD. 120528, by Envigo, Bresso, Italy) and a high sugar solution containing 23.1 g/L d-fructose and 18.9 g/L d-glucose (Merck-Sigma-Aldrich, Darmstadt, Germany) in addition to an intraperitoneal injection of CCl4 purchased by Merck-Sigma-Aldrich (Darmstadt, Germany) at a dose of 0.2 μL/g of body weight, once a week. The mice were harvested 96 h (hrs) after the last dose of CCl4. Body weight and food intake were measured every week. Liver weight was also measured at 12 weeks. At the end of the treatment, the animals were anesthetized (5% isofluorane) and blood and tissue samples were recovered and then sacrificed by using CO_2_ according to institutional guidelines. The blood samples were centrifuged at 4000 rpm for 10 min to obtain serum, which was then stored at −80 C for further analysis.

All the animal experiments were performed according to the guidelines of the Polytechnic University of Marche Institutional Animal Care and Use Committees (Protocol number 641/2019 and 40A31.1.EXT.1). Before and during the experimental procedure, all the animals were kept in the animal facility of the Polytechnic University of Marche at temperature of 20–24 °C with a 12-h light/12-h dark cycle. All aspects of the animal care and experimentation were performed following the Guide for the Care and Use of Laboratory Animals published by the Italian Laws (D.L.vo 116/92 and following additions), which enforces Directive EU86/609. Further details on the in vivo experiments can be found in the Supplementary Files.

### 2.2. Real-Time Quantitative PCR (QRT-PCR) in Mice

Total RNA was extracted from the mice’s liver and intestine using TRIzol^®^ reagent, following the manufacturer’s instructions (Thermo Fisher Scientific-Invitrogen, Waltham, MA, USA). Total RNA (1 μg) was converted to cDNA with random primers using the M-MLV Reverse Transcriptase (Promega, Milan, Italy). The mRNA level expression of target genes was determined using FAM-labeled probes purchased from Thermo Fisher Scientific-Applied Biosystems (Waltham, MA, USA). The mRNA levels were normalized to the endogenous control gene glyceraldehyde 3-phosphate dehydrogenase (GAPDH). Relative gene expression was calculated as 2 − ΔCt (ΔCt = Ct of the target gene minus Ct of GAPDH). The probes are listed in Supplementary Materials Table S1.

### 2.3. Tissue Histology

Isolated liver samples were fixed in 4% paraformaldehyde and embedded in paraffin. Next, the liver sections were stained using hematoxylin-eosin (H&E) and Masson’s trichrome. Steatosis was evaluated using a scale ranging from 0 to 3, as described by Brunt et al. [15]. The assessment of fibrosis was quantified by Masson’s trichrome and hydroxyproline quantification. Briefly, after staining with Masson’s trichrome (National Institutes of Health, Bethesda, MD, USA), ten different fields for each sample were examined to calculate the percentage of positive area using NIH ImageJ 1.54 g software. The results were then expressed as a fold change relative to the control. For the quantification of hydroxyproline, hepatic tissues were first homogenized and then precipitated with trichloroacetic acid. After incubation for 24 h at 110 °C in 6 N HCl and hydrolysis, the samples were analyzed using the Hydroxyproline Assay Kit (Sigma-Aldrich, St. Louis, MO, USA) and quantified by measuring the absorbance at 560 nm (Tecan Group Ltd., Männedorf, Switzerland).

### 2.4. ALT and AST Serum Levels Quantification

Plasma levels of alanine (ALT) and aspartate (AST) aminotransferase were assessed following the manufacturer’s protocol by using the Mouse ALT ELISA Kit and Mouse AST ELISA Kit purchased by Abcam (Cambridge, UK).

### 2.5. Hepatic Triglycerides Determination

The hepatic levels of triglyceride content were evaluated by using the Triglyceride Quantification Kit (Abcam, Cambridge, UK), following the manufacturer’s protocol. Briefly, 100 mg of liver tissue was homogenized in Nonidet-P40 at 5% (*v*/*v*) dissolved in H_2_O. The homogenate was then incubated for 5 min at 85 °C, followed by cooling to room temperature. The obtained samples were then centrifuged for 2 min to remove traces of insoluble material. Then, the supernatant was used for spectrophotometric analysis at a wavelength of 570 nm.

### 2.6. Immunofluorescence in Liver Tissues

The immunofluorescent (IF) staining was performed on paraffin-embedded liver tissues embedded by cutting 2-μm thick sections. Antigen retrieval was performed with ethylenediaminetetraacetic acid (EDTA) (pH 9) (Dako, Glostrup, Denmark). Next, liver sections were incubated overnight at 4 °C with the following primary antibodies: mouse monoclonal anti-CD68 antibody (Abcam, Cambridge, UK), rabbit polyclonal anti-CD206 antibody (Abcam, Cambridge, UK), rabbit polyclonal anti-SMAD2/3 antibody (Cell Signaling Technology, Inc., Danvers, MA, USA), and mouse monoclonal anti-PPARγ antibody (Santa Cruz Biotechnology Inc., Dallas, TX, USA). Then, after washing, samples were treated for one hour with labeled isotype-specific secondary antibodies, including 488AlexaFluor and 555AlexaFluor (Life Technologies-Invitrogen, Carlsbad, CA, USA). Nuclei were counterstained with Hoechst 33342. The images were acquired using the Olympus Fluoview FV3000 (Evident Europe GmbH, Olympus, Tokyo, Japan) confocal microscope, equipped with a laser combiner source (405 nm, 488 nm, 561 nm, 640 nm), and 60/1.42 oil objective. Optical single sections were acquired with a scanning mode format of 1024 × 1024 pixels, a sampling speed of 20 μs/pixel, and 12 bits/pixel images. Fluorochromes unmixing was performed by the acquisition of the automated-sequential collection of multi-channel images to reduce spectral crosstalk between channels. Background fluorescence signal of control cells stained only with the secondary antibodies conjugated to AlexaFluor-555. Representative images were captured and assembled using Adobe Photoshop CS6 software (Adobe Systems Inc., San Jose, CA, USA).

### 2.7. Lipopolysaccharide (LPS) Assay

In mice, LPS serum levels were assessed using a commercially available kit (Amebocyte Lysate (LAL) LAL Chromogenic Endpoint Assay (Hycult Biotech, Uden, The Netherlands).

### 2.8. Inflammatory Protein Array

For the evaluation of the serum expression levels of 40 different cytokines/chemokines (Supplementary Materials Table S2) in mice, the RayBio^®^ C-Series Mouse Inflammation Antibody Array C1 (RayBiotech, Inc., Norcross, GA, USA) was used, following the manufacturer’s protocol. The resultant chemiluminescence was analyzed by iBright Western Blot Imaging Systems (Thermo Fisher Scientific-Invitrogen, Waltham, MA, USA) and quantified by calculating the integrated density with Image J v3.91 software.

### 2.9. RNA Extraction and Analysis of Inflammatory Genes

The extraction of total RNA was performed using Norgen’s Total RNA Isolation Plus Kit (Norgen Biotek Corp, Thorold, ON, Canada), and its quantification was assessed using NanoDrop Technologies (Wilmington, DE, USA). Next, 2 μg of total RNA were reverse transcribed using SuperScript™ VILO™ cDNA Synthesis Kit (Thermo Fisher Scientific-Invitrogen, Waltham, MA, USA). Then, cDNA was used to quantitatively analyze the expressions of 632 genes involved in inflammatory and immune response and 16 endogenous housekeeping genes. In particular, the analysis was carried out using TaqMan^®^ OpenArray^®^ Real-Time PCR Master mix and TaqMan^®^ OpenArray^®^ Mouse Inflammation (Thermo Fisher Scientific-Applied Biosystems, Waltham, MA, USA). The TaqMan^®^ probes in the array are listed in Supplementary Materials Table S3. Gene expression was quantified as a relative quantity (RQ) with respect to GAPDH and the respective controls using Thermo Fisher Cloud Resources. Then, the mean of RQ was analyzed by the quantile binning algorithm in CIMminer (http://discover.nci.nih.gov/cimminer, accessed on 20 February 2023) and plotted as a Heatmap.

### 2.10. Determination of Serum and Hepatic Total Cholesterol and Lipoproteins

Reported in the Supplementary File.

### 2.11. Cell Culture Experiments

The description of cell culture models, treatments, and analyses can be found in the Supplementary File.

### 2.12. Statistics

The data are presented as means ± standard deviation (SD) from at least two independent experiments repeated in duplicate. All the statistical analyses were performed using the two-sided student’s t-test or Anova P by using GraphPad Prism 9.0 (GraphPad Software, San Diego, CA, USA). Values of *p* < 0.05 were considered to be statistically significant.

## 3. Results

### 3.1. Constitutive Intestinal Activation of LXRα Reduces WD/CCl4-Dependent Liver Weight and Liver Function

As already described [15], WD associated with the injection of low doses of CCl4 induces weight gain and MASLD in animal models. Therefore, to determine the potential effect of the intestinal activation of LXRα on obesity and MASLD, WT and iLXRα mice were fed an ND or a diet combining WD with CCl4 (WD/CCl4), as previously described by Tsuchida et al. [16]. As expected, diets and VP16LXRα transgene expression significantly affected LXRα gene expression only at the intestinal level (Figure S1A,B in the Supplementary Materials). Moreover, confirming the iLXRα activation in transgenic mice, gene expression of both transporters ABCG5 and ABCG8 was unaffected by diet at the liver level but significantly increased in the intestine of transgenic mice (Figure S1C–F in the Supplementary Materials).

**Figure 1:**
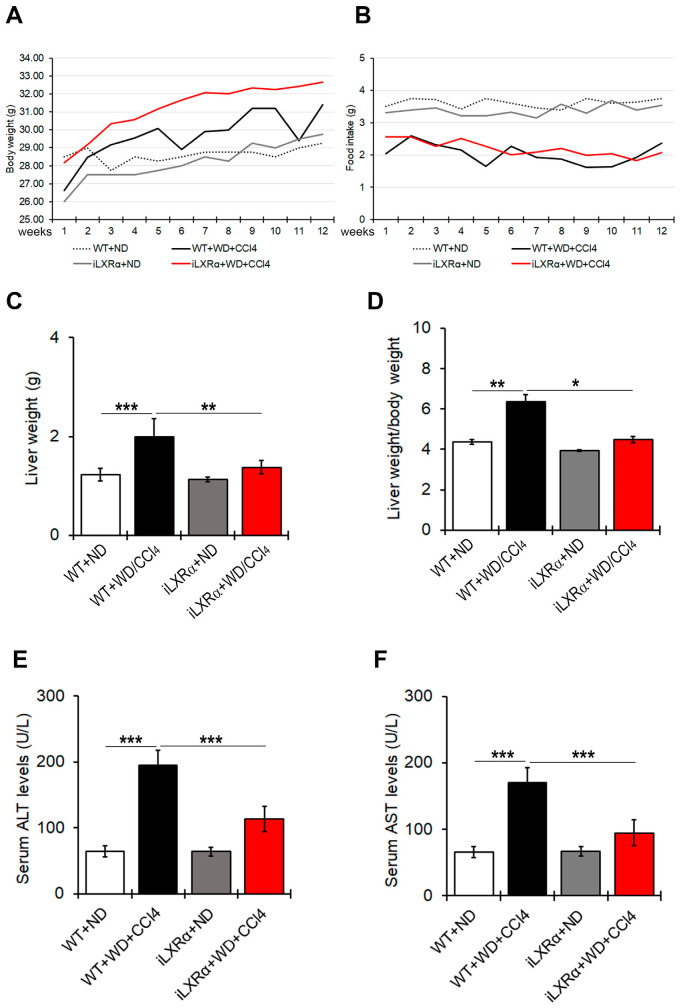
Effect of constitutive intestinal activation of LXRα in WD/CCl4-treated mice on body weight, food intake, liver weight, and liver-to-body weight ratio. The graphs show changes in (**A**) body weight and (**B**) food intake over 12 weeks in WT and iLXRα mice treated with ND and WD/CCl4. The bar graphs report (**C**) liver weight, (**D**) liver weight/body weight ratio, (**E**) serum ALT levels and (**F**) serum AST levels at the end of treatment. Data are expressed as the mean ± SD of at least *n* = 5 animals per group * *p* < 0.05, ** *p* < 0.01 and *** *p* < 0.001.

As shown in Figure 1A,B, WT and iLXRα mice treated with WD/CCl4 exhibited body weight gain despite reduced food intake compared to their control mice treated with ND. After 12 weeks of treatment, liver and liver-to-body weight ratios in iLXRα+ND mice were equal to control WT+ND mice, while their increase due to WD/CCl4 in WT mice was significantly reduced in iLXRα+WD/CCl4 mice (Figure 1C,D).

Serum ALT and AST levels were higher in WT+WD/CCl4 than in WT+ND mice, highlighting a liver injury that was significantly less evident in iLXRα+WD/CCl4 mice (Figure 1E,F).

### 3.2. Constitutive Intestinal Activation of LXRα Reduces WD/CCl4-Dependent Liver Weight and Steatosis

**Figure 2:**
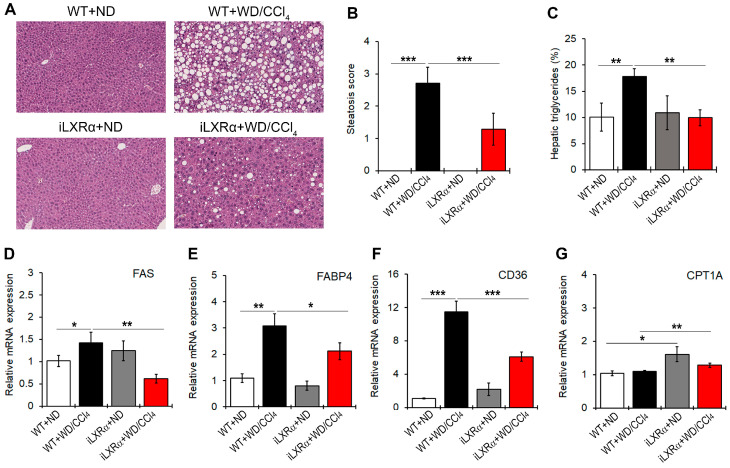
Effect of constitutive intestinal activation of LXRα in WD/CCl4-treated mice on histological and biochemical parameters in liver tissue. (**A**) Representative H&E staining for liver tissues in WT and iLXRα mice treated with ND and WD/CCl4 (20× magnification). The bar graphs report (**B**) the histological steatosis scores; (**C**) the levels of hepatic triglycerides; and hepatic mRNA expression of FAS (**D**); FABP4 (**E**); CD36; (**F**) and CPT1A (**G**) genes. Gene expression is reported as a relative expression by QRT-PCR normalized to GAPDH transcript levels. Data are expressed as the mean ± SD of at least *n* = 5 animals per group. * *p* < 0.05, ** *p* < 0.01 and *** *p* < 0.001.

In our model, iLXRα-affected liver steatosis in the ND condition, whereas it protected mice from developing hepatic steatosis under WD/CCl4 treatment (Figure 2A,B). Consistently, hepatic levels of triglycerides were higher in WT+WD/CCl4 mice than in iLXRα+WD/CCl4 mice (Figure 2C). These macroscopic effects were confirmed by evaluating the expression of lipogenic genes. In particular, no changes were observed when iLXRα+ND mice were compared with WT+ND mice, whereas the increased expression of FAS, FABP4, and CD36 genes observed in WT+WD/CCl4 was lowered in iLXRα+WD/CCl4 mice (Figure 2D–F). The anti-lipogenic effect of the LXRα intestinal activation in the liver was also corroborated by the significant upregulation of the rate-limiting enzyme for long-chain fatty acid beta-oxidation CPT1A (Figure 2G).

Moreover, even if WD/CCl4 treatment had no effect on the expression of the CPT1A gene in WT mice, intestinal activation of LXRα up-regulated CPT1A transcript. 

### 3.3. Constitutive Intestinal Activation of LXRα Reduces WD/CCl4-Dependent Inflammation in Mice

**Figure 3:**
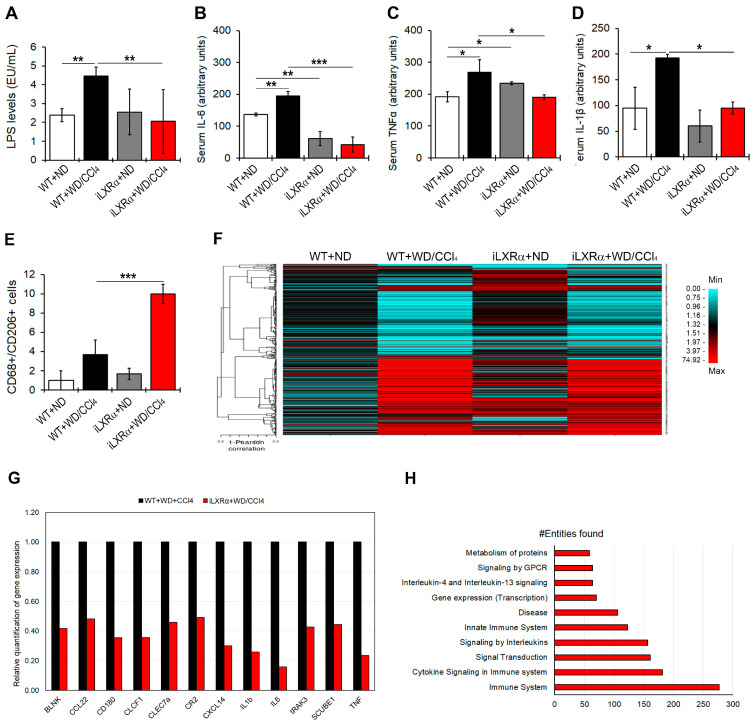
Effect of constitutive intestinal activation of LXRα in WD/CCl4-treated mice on systemic and hepatic inflammation. The bar graphs show quantification of (**A**) LPS; (**B**) IL-6; (**C**) TNFα; and (**D**) IL-1β serum levels in WT and iLXRα mice treated with ND and WD/CCl4. (**E**) Number of hepatic CD68+/CD206+ cells by immunostaining; (**F**) Heatmap shows the Pearson correlation matrix of 382 hepatic inflammatory genes significantly changed among groups; (**G**) Bar graphs show the relative expression of the significantly downregulated pro-inflammatory genes in iLXRα+WD/CCl4 respect to WT+WD/CCl4 considered as 1; (**H**) Bar plot of the 10 most abundant pathways (by Reactome) in which restored genes were involved. Data are expressed as the mean ± SD of at least *n* = 5 animals per group. * *p* < 0.05, ** *p* < 0.01 and *** *p* < 0.001.

As shown in Figure 3A, WT+WD/CCl4 mice exhibited increased LPS serum levels compared to control mice, whereas the intestinal activation of LXRα in mice exposed to WD/CCl4 treatment resulted in lower LPS serum levels than in WT+WD/CCl4 mice.

When a pool of 40 circulating inflammatory signals was analyzed by protein array, the intestinal activation of LXRα was able to counteract only the WD/CCl4-dependent upregulation of pro-inflammatory cytokines, IL-6, TNFα, and IL-1β (Figure 3B–D and Supplementary Materials Figure S2).

Next, we evaluated the hepatic inflammatory status. As reported in Figure 3E and Supplementary Materials Figure S3, the number of CD68+/CD206+ positive cells, representing a measure of M2 macrophages, was significantly reduced in WT+WD/CCl4 compared to WT+ND mice but strongly recovered in mice with the intestinal activation of LXRα. These data were supported by analyzing the hepatic expression of 632 genes that could be affected in inflammatory diseases. Among these, 382 genes exhibited changes in gene expression among the experimental groups (Figure 3F and Supplementary Materials Table S4). Of note, 153 genes were significantly upregulated in WT+WD/CCl4 compared to WT+ND mice, but only 12 of them were significantly reduced in iLXRα+WD/CCl4 mice compared to WT+WD/CCl4 mice (Figure 3G). Reactome analysis highlighted that these genes are involved in inflammatory pathways, signal transduction, and gene expression control (Figure 3H).

### 3.4. Constitutive Intestinal Activation of LXRα Reduces WD/CCl4-Dependent Fibrosis in Mice

**Figure 4:**
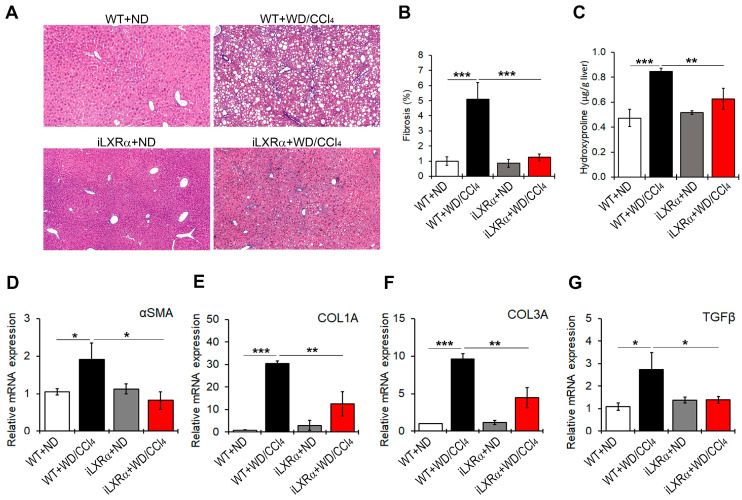
Effect of constitutive intestinal activation of LXRα in WD/CCl4-treated mice on hepatic fibrogenesis. (**A**) Representative liver sections stained with Masson’s trichome in ND and WD/CCl4 treated WT and iLXRα mice (20× magnification). The bar graphs report (**B**) the histological score and (**C**) the liver hydroxyproline content. Relative mRNA expression of hepatic genes involved in fibrogenesis, including (**D**) αSMA; (**E**) COL1A; (**F**) COL3A; and (**G**) TGFβ. Gene expression is reported as a relative expression by QRT-PCR normalized to GAPDH transcript levels. Data are expressed as the mean ± SD of at least *n* = 5 animals per group. * *p* < 0.05, ** *p* < 0.01 and *** *p* < 0.001.

WD/CCl4 treatment significantly induced liver fibrosis (Figure 4A,B) and increased collagen fibers (Figure 4C) in WT mice, but not in iLXRα mice. Consistently, the hepatic gene expression of fibrosis markers αSMA, COL1A, COL3A, and TGFβ was significantly upregulated in WD/CCl4 WT mice compared to ND-fed WT mice (Figure 4D–G). However, under intestinal LXRα activation, the upregulation of hepatic fibrosis markers was significantly inhibited. As reported in Figure 4D–F, the intestinal activation of LXRα restored the αSMA and TGFβ mRNA expression levels to those observed in WT mice and attenuated the hepatic gene expression of COL1A and COL3A.

### 3.5. Constitutive Intestinal Activation of LXRα Decreases WD/CCl4-Dependent Cholesterol Absorption and Induces Reverse Cholesterol Transport (RCT)

**Figure 5:**
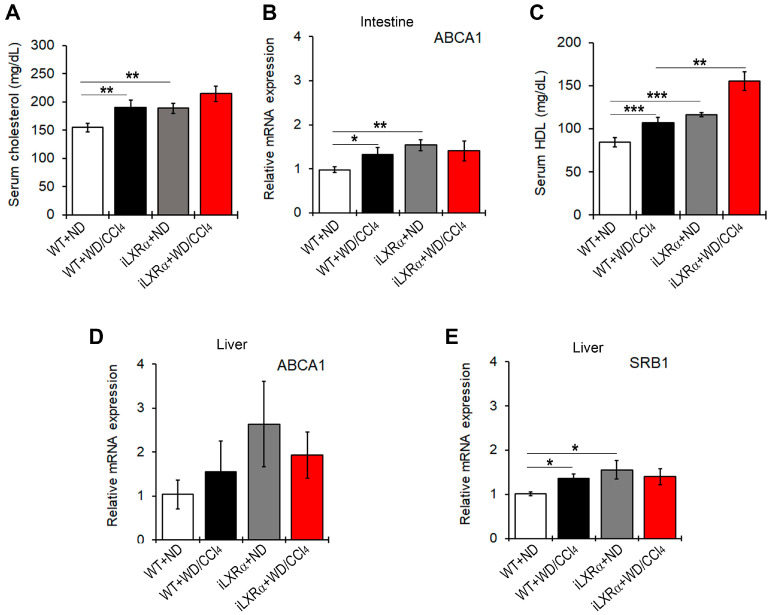
Effect of constitutive intestinal activation of LXRα in WD/CCl4-treated mice on cholesterol efflux and RCT. The bar graphs show (**A**) quantification of total cholesterol serum levels; (**B**) relative mRNA expression of the ABCA1 gene in the intestine; (**C**) quantification of the HDL serum levels; and the relative mRNA expression of (**D**) ABCA1 and (**E**) SRB1 in the liver of WT and iLXRα mice treated with ND and WD/CCl4. Gene expression is reported as a relative expression by QRT-PCR normalized to GAPDH transcript levels. Data are expressed as the mean ± SD of at least *n* = 5 animals per group. * *p*<0.05, ** *p* < 0.01 and *** *p* < 0.001.

Next, we found that total serum cholesterol levels were increased in WT and iLXRα mice treated with WD/CCl4 compared to those fed ND (Figure 5A). These data were supported by changes in ABCA1 gene expression at the intestinal level (Figure 5B). However, mice with iLXRα exhibited a more significant increase in HDL serum levels compared to WD/CCl4 (Figure 5C).

At the hepatic level, there were no statistically significant changes in ABCA1 gene expression (Figure 5D), whereas significant changes were observed in SRB1 levels. The expression levels of the SRB1 gene were higher in WD/CCl4-treated WT mice and in iLXRα mice than in WT mice (Figure 5E), even if the hepatic levels of total cholesterol and HDL in transgenic mice were significantly lower than in WT-treated mice (Supplementary Materials Figure S4A,B).

### 3.6. SRB1 Mediates the HDL-Dependent Anti-Steatogenic Effects In Vitro

**Figure 6:**
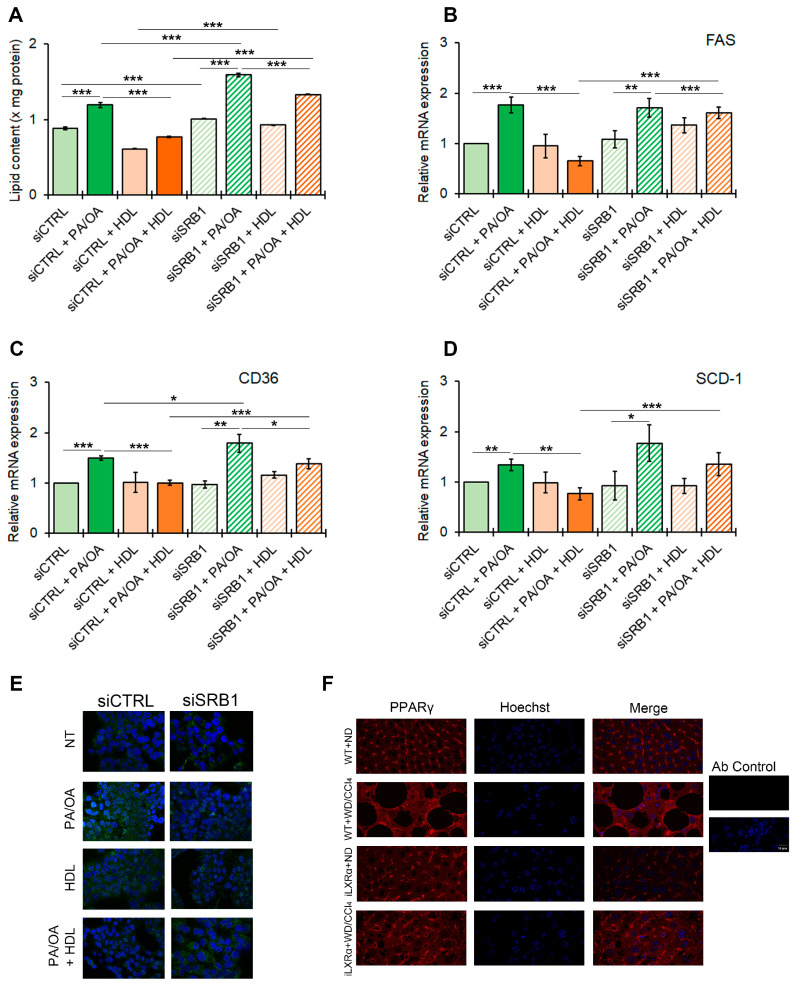
Effect of SRB1 silencing on lipid metabolism and downstream signaling. The bar graphs show (**A**) quantitative lipid content by ORO staining; mRNA expression of (**B**) FAS; (**C**) CD36; and (**D**) SCD-1. Gene expression is reported as relative expression by QRT-PCR vs. siCTRL normalized to GAPDH transcript levels. Data are expressed as the mean ± SD of at least *n* = 3 independent experiments. * *p* < 0.05, ** *p* < 0.01 and *** *p* < 0.001. (**E**) representative cropped confocal imaging by IF (Magnification 60×) of the merge between PPARγ (green) and nuclei-Hoechst (blue) in siCTRL and siSRB1 HepG2 cells treated with FFAs (PA/OA) alone or in combination with pre-exposure to HDL and collected after 24 h; (**F**) Representative confocal imaging by IF of PPARγ (red), nuclei-Hoechst (blue) and merge in liver tissue from WT and iLXRα mice treated with ND and WD/CCl4 (Magnification 60×).

Our data suggest that the iLXRα-dependent increase in circulating HDL levels may be associated with the protective effects observed in a MASH animal model. To test the role of SRB1 as a plausible mediator of HDL-related anti-steatogenic and anti-inflammatory effects, we next established HepG2 control cells (siCTRL) and HepG2 cells silenced for SRB1 (siSRB1). The knockdown efficiency resulted in a 60% and 70% reduction at the mRNA and protein levels, respectively (Supplementary Materials Figure S5A,B). As reported in Figure 6A, the treatment with HDL reduced intracellular lipids in HepG2 cells after stimulation with FFAs, and this effect was inhibited by SRB1 depletion. This pattern of lipid changes was supported by the impact on the expression of genes involved in lipid metabolism, including FAS, CD36, and SCD-1 (Figure 6B–D). It is well known that all these genes in hepatocytes are controlled by PPARγ in the presence of a lipid overload [17]. As shown in Figure 6E and Supplementary Materials Figure S6, FFAs enhanced protein expression and nuclear translocation of PPARγ in siCTRL HepG2, an effect that was inhibited by HDL treatment. In contrast, SRB1 depletion abolished the HDL-dependent impairment of PPARγ nuclear translocation associated with FFAs.

As in vitro models, mice with MASLD (WT+WD/CCl4 mice) showed an increased nuclear translocation of PPARγ, while in mice with the intestinal activation of LXRα (iLXRα+WD/CCl4 mice), in which, as reported above, the rise in serum HDL is coupled with a reduced steatogenic effect, we observed a reduction in PPARγ nuclear translocation (Figure 6F).

### 3.7. SRB1 Mediates the HDL-Dependent Anti-Fibrotic Effects In Vitro

**Figure 7:**
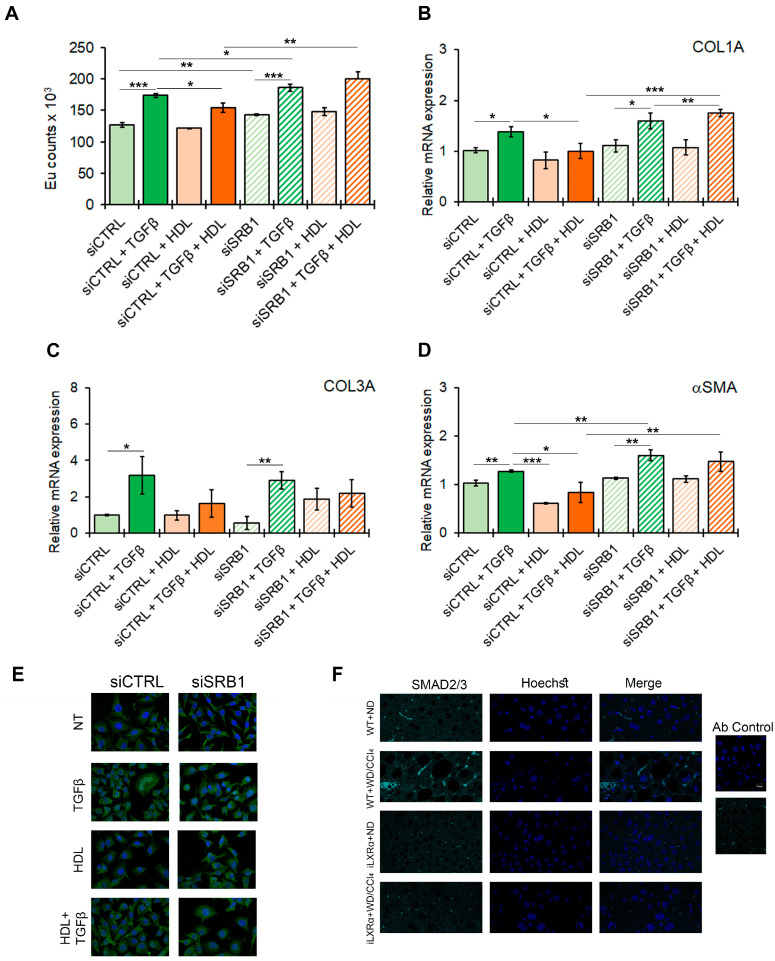
Effect of SRB1 silencing on fibrogenesis and downstream signaling. The bar graphs show (**A**) cell proliferation evaluated by a BrdU incorporation assay and expressed as Eu counts; mRNA expression of (**B**) COL1A; (**C**) COL3A; and (**D**) αSMA. Gene expression is reported as a relative expression by QRT-PCR vs. siCTRL normalized to GAPDH transcript levels. Data are expressed as the mean ± SD of at least *n* = 3 independent experiments. * *p* < 0.05, ** *p* < 0.01 and *** *p* < 0.001. (**E**) representative cropped confocal imaging by IF (Magnification 60×) of the merge between SMAD2/3 (green) and nuclei-Hoechst (blue) in siCTRL and siSRB1 LX-2 cells treated with TGFβ alone or in combination with pre-exposure to HDL and collected after 24 h. (**F**) Representative confocal imaging by IF of SMAD2/3 (cyan), nuclei-Hoechst (blue) and merge in liver tissue from WT and iLXRα mice treated with ND and WD/CCl4 (Magnification 60×).

To confirm the potential effect of the HDL-SRB1 axis on liver fibrosis, as hypothesized from the in vivo data, we tested the effect of SRB1 silencing in HSC LX-2 cells exposed to TGFβ as a profibrogenic stimulus. The efficiency of SRB1 gene silencing is reported in Supplementary Materials Figure S7A,B. As shown in Figure 7A, treatment with HDL counteracts the TGFβ-induced HSC activation, returning the proliferation rate to control levels (Figure 7A) and restoring the expression of profibrogenic markers, including COL1A, COL3A, and αSMA (Figure 7B–D). SRB1 depletion abolished the antifibrotic effect of HDL, as shown by the data on HSC LX-2 cell proliferation and COL1A and αSMA gene expression.

Moreover, in LX-2 cells, the TGFβ-mediated nuclear translocation of SMAD2/3, a key factor involved in intracellular signaling of TGFβ, was abolished by serum HDL, and this effect was abolished by the silencing of SRB1 (Figure 7E and Supplementary Materials Figure S7C). Finally, SMAD2/3 nuclear translocation was evident in WT+WD/CCl4 mice, while this effect was reduced in iLXRα+WD/CCl4 mice (Figure 7F).

## 4. Discussion

MASLD has become a significant global public health problem in recent years [3]. MASLD is a progressive condition that can be reversed by lifestyle intervention at the simple steatosis stage. Still, for MASH, its progressive form is characterized by hepatocellular ballooning, necro-inflammation, and fibrosis, and approved treatments are poor [18]. Therefore, several lines of experimental evidence have suggested that LXR activation may be essential for treating metabolic disorders, including those associated with MASLD, making it an attractive pharmacological target in humans [19,20,21,22].

Since the LXRs have emerged as crucial regulators of lipogenesis, glucose metabolism, and, most importantly, cholesterol efflux and transport, several different types of LXR modulators have been designed over the last 30 years, including synthetic LXRα agonists. Among them, LXR-623, GW3965, GSK9772, and BMS-779788 have been evaluated in clinical trials, showing improvements in hypercholesterolaemia and atherosclerosis [23]. Despite these suitable premises, the development of LXR agonists as therapeutic agents has been hindered by various side effects, including increased hepatic lipogenesis and consequent steatosis, hypertriglyceridemia, and neuroinflammation, most of which are attributed to systemic and/or liver-specific activation of LXRα [24]. Moreover, the evidence of LXR upregulation of lipogenic enzymes SREBP1c, FASN, and SCD1, and the subsequent promotion of lipogenesis in experimental models, has hampered the clinical use of these agonists, especially in the treatment of MASLD [19].

To overcome this issue, the specific and selective extra-hepatic activation of LXRα could provide an alternative strategy. Indeed, it has already been demonstrated that intestinal activation of LXRα can reduce cholesterol absorption and increase HDL production, thereby reducing circulating cholesterol without any LXR-dependent steatogenic effects [14]. Concerning hepatic injury, we have recently demonstrated that the specific and selective intestinal activation of LXRα reduced oxidative stress, inflammation, and fibrosis in CCl4-treated mice [13].

In this study, we demonstrated the protective role of the specific and selective intestinal activation of LXRα receptor against the progression of liver damage in a MASLD/MASH model. WT mice and iLXRα mice, carrying constitutive intestinal activation of the α isoform of the LXR receptor, were treated with CCl4 and WD to reproduce the histological features of MASH [16]. Our data revealed that intestinal activation of LXRα in the MASH model counteracted the body and liver weight gain and improved the histological features of MASH induced by the combination of WD and CCl4. Intestinal activation of LXRα may protect against hepatic steatosis by promoting the restoration of liver triglyceride content through modifications in the hepatic expression of genes involved in lipid synthesis, storage, and uptake. We identified the HDL/SRB1 axis as a critical promoter of HDL-dependent reversal effects on hepatocellular lipid accumulation and profibrogenic phenotype in HSCs. In fact, the LXR/RXR system is one of the regulators of SRB1 promoter transcription in the liver [25], but the mechanism by which the intestinal activation of LXRα may cause increased expression of SRB1 in the liver is not explored in our study. Piccinin et al. [26] recently demonstrated that the intestinal LXR may play a role in the hepatic cholesterol metabolism and SRB1 in a model of MASH-related HCC, suggesting that the specific activation of LXR axis in the gut may induce changes in the enteropathic circulation (e.g., enterokines and/or metabolites), thus modifying transcriptional regulation in distant organs, such as the liver. This could be a point that warrants further investigation in the future.

However, the effects of LXRα activation were not limited to lipid metabolism and fibrosis. Indeed, in vivo data have highlighted a significant intrahepatic anti-inflammatory effect of LXRα, although the molecular-level mechanism remains unclear [13,27]. Similarly, we found that key mediators of both hepatic and extra-hepatic clinical manifestations related to MASH were reversed by intestinal activation of LXRα. In particular, we observed a switch in macrophage phenotype from pro-inflammatory to anti-inflammatory in liver tissues from WD/CCl4 animals, accompanied by intestinal activation of LXRα. This was associated with a downregulation of several circulating and hepatic pro-inflammatory cytokines and chemokines, which require further exploration.

Furthermore, we demonstrated that. in our model, the intestinal activation of LXRα favored RCT, mainly by increasing circulating and hepatic HDL cholesterol levels without perturbing the expression of LXR target genes in the liver, such as ABCG5 and ABCG8. These findings were in line with those of Lo Sasso et al. [14], thus corroborating the critical role of intestinal-specific LXRα activation in regulating lipid and cholesterol homeostasis without producing side effects, such as liver steatosis and increased fatty acid synthesis. Our findings on the beneficial role of intestinal LXRα are consistent with the effect of a gut-specific LXR agonist, GW6340, an ester synthesized from GW3965 that has been shown to selectively induce the expression of LXR target genes in the intestine, without causing changes in the expression of genes involved in lipogenesis in the liver [28].

All our findings support the evidence that the rise in HDL levels occurring in the context of intestinal LXRα activation may exert beneficial effects on MASH-associated liver damage via a functional cellular response to SRB1, which may influence both liver parenchymal and non-parenchymal cells.

These protective effects of HDL on MASH were previously reported in the Pioglitazone versus Vitamin E versus Placebo for the Treatment of Nondiabetic Patients with Non-alcoholic Steatohepatitis (PIVENS) trial, where the prominent role of the PPARγ agonist pioglitazone in the elevation of HDL levels was reported. In contrast, PPARα/γ agonists have been shown to improve MASH-related liver damage in patients [29,30]. Moreover, previous evidence in mice has demonstrated that SRB1 whole-body deficiency decreases the activity of PPARγ in adipose tissue, thereby impairing the flux of fatty acids from adipose tissue to the liver [29]. Accordingly, our in vitro and in vivo data provided definitive insights into the role of the hepatic SRB1/PPARγ axis in MASH development. Indeed, our data revealed that HDL stimulation of fatty HepG2 cells reduced the nuclear translocation of PPARγ, resulting in the downregulation of lipid accumulation and the expression of steatogenic genes. Moreover, in agreement with Rivera et al. [30], silencing SRB1 impaired the HDL-dependent beneficial effects on PPARγ nuclear translocation and steatosis patterns.

As already described by Pierantonelli et al. [13], the selective intestinal activation of LXRα reduced chemical-induced fibrosis; in this study, we reinforced this concept by demonstrating a similar effect in MASH mice. Moreover, our in vitro results also demonstrated that the HDL/SRB1 axis promotes the disruption of SMAD2/3 protein localization in HSC cells, thereby blocking the TGFβ/SMAD2/3 signaling and preventing the fibrogenic phenotype in HSCs. Indeed, even if Lin et al. [31] reported that HDLs counteracted the endothelial-mesenchymal transition in endothelial progenitor cells by controlling the TGFβ-dependent phosphorylation of SMAD2/3 proteins, this pathway had not yet been associated with the expression SRB1. Our findings reinforce the potential therapeutic role of molecules targeting TGFβ/SMADs signaling that, even if explored in models, needs more data in clinical studies [32]. The definition of this new pathogenetic pathway supports the current strategy in designing clinical trials based on LXR inverse agonists that, by suppressing the transcriptional activity of LXRα, may reduce the expression of lipogenic enzymes and consequent fat accumulation in the liver, as reported, by the administration of SR9238 to diet-induced murine models [33]. Still, developing novel targeted pharmacological or genetically centered (i.e., RNA-based) approaches with a focus on SRB1 may improve liver damage occurring in MASLD [33,34]. However, the hepatotoxic effect of inverse LXR agonists or genetic-based strategies requires further evidence before being clinically translated and applied to MASLD and dyslipidaemia treatment.

## 5. Conclusions

In conclusion, our results demonstrate that selective intestinal LXRα activation, the consequent increase in HDL levels, and hepatic modulation of SRB1 play a key role in regulating MASH progression by modulating PPARγ- and/or TGFβ/SMADs-dependent mechanisms.

## Data Availability

The original contributions presented in this study are included in the article/Supplementary Materials; further inquiries can be directed to the corresponding author.

## Supplementary Materials

The following supporting information can be downloaded at: https://www.mdpi.com/article/10.3390/nu17081349/s1, Table S1: Probes for QRT-PCR in mice; Table S2: Antibody array MAP; Table S3: TaqMan^®^ probes for QRT-PCR Open Array; Table S4: Expression data of inflammatory genes dysregulated genes in WD/CCl4 treated mice with or without intestinal activation of LXRα; Figure S1: Effect of constitutive intestinal activation of LXRα in WD/CCl4 treated mice on intestinal and hepatic control of cholesterol absorption; Figure S2: Effect of constitutive intestinal activation of LXRα in WD/CCl4 treated mice on circulating cytokines and chemokines; Figure S3: Effect of constitutive intestinal activation of LXRα in WD/CCl4 treated mice on hepatic expression of macrophages markers; Figure S4: Effect of constitutive intestinal activation of LXRα in WD/CCl4 treated mice on hepatic cholesterol and HDL content; Figure S5: The effect of SRB1 silencing on the expression of SRB1 transcript and protein in HepG2 cells after 24 h from silencing; Figure S6A: Single staining in the image of panel E in Figure 6; Figure S6B: Uncropped image of panel E in Figure 6; Figure S7: The effect of SRB1 silencing on the expression of SRB1 transcript and protein in LX2 cells after 24 h from silencing. Reference [35] is cited in the supplementary materials.

## Abbreviations

The following abbreviations are used in this manuscript:

αSMA	alpha smooth muscle actin
ABCA1	ATP-Binding Cassette Transporter 1
ABCG	ATP-binding cassette transporters G
ALT	alanine aminotransferase
AST	aspartate aminotransferase
CCl4	Carbon tetrachloride
CD206	Mannose receptor C-type 1
CD36	Thrombospondin receptor
CD68	Type I transmembrane glycoprotein
COL	Collagen
CPT1A	Carnitine Palmitoyltransferase 1A
FABP4	Fatty Acid Binding Protein 4
FAS	Fatty acid synthase
GAPDH	Glyceraldehyde 3 Phosphate Dehydrogenase
H&E	Haematoxylin-eosin
HDL	High-density lipoprotein
IL	Interleukin
iLXRα	Intestinal Liver X receptors alpha
LDL	Low-density lipoprotein;
LPS	Lipopolysaccharide
LXRs	Liver X receptors
MASH	Metabolic dysfunction-associated steatohepatitis
miRNAs	Small noncoding microRNAs
NAFLD	Non-alcoholic fatty liver disease
MASLD	Metabolic dysfunction-associated steatotic liver disease
ND	Normal diet
PPAR	Peroxisome proliferator-activated receptors gamma SMAD2/3
RCT	Reverse cholesterol transport
SMAD2/3	Small mothers against decapentaplegic homolog protein proteins 2/3
SRB1	Scavenger receptor class B type 1
T2D	Type 2 diabetes
TGFβ	Transforming Growth Factor-Beta
TNFα	Tumor necrosis factor-α
VLDL	Very low-density lipoprotein
WD	Western diet
WT	Wild-type
