# Supplementary material for: Intestinal Activation of LXRα Counteracts Metabolic-Associated Steatohepatitis Features in Mice
Source: Nutrients. 2025 Apr 15;17(8):1349. doi: 10.3390/nu17081349 (PMC12030714; doi:10.3390/nu17081349)
Supplement: Supplementary file 1 [file nutrients-17-01349-s001.zip › nutrients-3536659-supplementary.pdf]

Supplementary material

Intestinal activation of LXRα counteracts metabolic-associated steatohepatitis features in mice

Gessica Lioci [1,†], Fabio Gurrado [1,†], Nadia Panera [2,†], Marzia Bianchi [2], Cristiano De Stefanis [3], Valentina D'Oria [3], Nicolò Cicolani [3], Silvano Junior. Santini [4], Laura Schiadà [1], Anna Alisi [2,*] and Gianluca Svegliati-Baroni [1]

[1] Liver Injury and Transplant Unit, Polytechnic University of Marche, 60121 Ancona, Italy; gessicalioci@gmail.com (G.L.); gurrado.fabio@gmail.com (F.G.); laura.schiada@ospedaliriuniti.marche.it (L.S.); gsvegliati@gmail.com (G.S.-B.)
[2] Research Unit of Genetics of Complex Phenotypes, Bambino Gesù Children's Hospital, IRCCS, 00165 Rome, Italy; nadia.panera@opbg.net (N.P.); marzia.bianchi@opbg.net (M.B.); anna.alisi@opbg.net (A.A.)
[3] Core Facilities, Bambino Gesù Children's Hospital, IRCCS, 00146 Rome, Italy; cristiano.destefanis@opbg.net (C.D.S.); valentina.doria@opbg.net (V.D.); nicolo.cicolani@opbg.net (N.C.)
[4] Department of Life, Health and Environmental Sciences-MESVA, School of Emergency-Urgency Medicine, University of L'Aquila, 67100 L'Aquila, Italy; silvanojunior.santini@univaq.it (S.J.S)
* Correspondence: anna.alisi@opbg.net
† These authors equally contribute to the manuscript.

TABLE OF CONTENTS

Experimental Section

Supplementary Tables

Supplementary Figures

References

Experimental section

Determination of serum total cholesterol and lipoproteins

The analysis of total cholesterol, high-density lipoprotein (HDL), low-density lipoprotein (LDL) and very low-density lipoprotein (VLDL) was performed in serum samples by using the HDL and LDL/VLDL Cholesterol Assay kit (Abcam, Cambridge, UK) following the manufacturer's protocol. The subclasses of lipoproteins were separated upon centrifugation, and absorbance was measured at 560 nm (Tecan Group Ltd., Männedorf, Switzerland).

Determination of hepatic cholesterol and lipoproteins

Total liver cholesterol was assayed using the Cholesterol Fast kit (Diacron Labs, Grosseto, Italy). Briefly, 10 mg of liver tissue was homogenized with 200 μL of Chloroform: isopropanol:NP-40 at a 7:11:0.1 ratio, and centrifuged at 15,000 × g. To remove the chloroform, the supernatant was then subjected to evaporation at 50°C. The resultant product, after a series of enzymatic reactions, was quantified at a wavelength of 505 nm. The levels of hepatic HDL were determined using the Mouse HDL ELISA Kit (Elabscience, Wuhan, China) according to the manufacturer's suggested protocol.

Cell culture and reagents

Human HepG2 cells purchased from ATCC (Manassas, Virginia, USA) and human LX-2 cells were purchased by Merck-Sigma-Aldrich (Darmstadt, Germany) with included certification. All cell lines were grown in Dulbecco modified Eagle medium supplemented with 10% Eagle Medium (DMEM) supplemented with 10% fetal bovine serum (FBS) and 1% penicillin and streptomycin (Thermo Fisher Scientific-Gibco, Waltham, MA, USA) at 37 ◦C in a humidified atmosphere of 5% CO2 and 95% air. Before the experiments, LX-2 cells were pre-cultured in DMEM supplemented with 1% FBS for 12 h. Opti-MEM was purchased from Invitrogen (Carlsbad, CA, USA). INTERFERin was obtained by Polyplus-transfection S.A (Illkirch, France), Silencer Pre-designed siRNAs for the scavenger receptor class B type 1 (siSRB1) and Silencer Select Negative Control (siCTRL) were purchased by Ambion Life Technologies Corporation (Woburn, MA). HDL (Sigma-Aldrich, Saint Louis, MO), Oleic acid (OA), palmate (PA) and Oil red O (ORO) were obtained from Sigma-Aldrich (Munich, Germany).

Cell transfection and treatments

HepG2 and LX-2 cells were transfected with SRB1 or scrambled siRNA, serving as a negative control. Cells were plated in 12-well culture plates at a density of 250.000 cells/well. Transfection was performed with 30 nM shRNA using INTERFERin reagent according to the INTERFERin in vitro siRNA Transfection Protocol (Polyplus, Illkirch, France). After 24 hours from silencing, HepG2 cells siSRB1 and siCTRL were or not pre-incubated for 1 hour with 1 mg/ml HDL (Sigma-Aldrich, Saint Louis, MO), then cells were exposed to free fatty acids (FFAs) mixture (OA and PA in molar ratio 3:1) 0.5 mM for 24 hours. While siCTRL and siSRB1 LX-2 cells were or were not pre-incubated for 1 hour with HDL (500 ug/ml) and then activated with 10 ng/mL tumour growth factor (TGF)-β for 24 h. After 24 hours the cells were used for quantitation of lipid content ORO as previously described [13].

QRT-PCR in cells

Total RNA was isolated from control (siCTRL) and silenced (siSRB1) HepG2 and LX-2 cell lines, untreated and treated with HDL (1 mg/ml) and OA+PA and HDL (1 mg/ml) and TGF-β, respectively, as described in the Transfection section. RNA purification plus kit (Norgen Biotek Corp, Thorold, ON, Canada) was used for total RNA purification. Genomic DNA was digested using a genomic DNA removal kit (Norgen Biotek Corp, Thorold, ON, Canada). The mRNA expression level of target genes was determined using FAM-labeled probes purchased from Thermo Fisher Scientific (Thermo Fisher, Waltham, MA, USA). The mRNA levels were normalized to the endogenous control gene glyceraldehyde 3-phosphate dehydrogenase (GAPDH). Relative gene expression was calculated as 2−ΔCt (ΔCt = Ct of the target gene minus Ct of GAPDH). Probes are listed in **Table S1.**

Western blotting

Control (siCTRL) and silenced (siSRB1) HepG2 and LX-2 cells were cultured as described in "Cell Transfection and Treatment." Cells were harvested using a cell scraper and cold PBS, and then lysed in RIPA buffer (Merck-Sigma-Aldrich, Darmstadt, Germany) containing protease inhibitors and phosphatase inhibitors (Thermo Fisher Scientific-Pierce, Waltham, MA, USA). The lysates were centrifuged at 12,000 rpm for 15 min at 4°C, and then the supernatants were collected. Total protein sample concentrations were measured by using a BCA assay kit by Thermo Fisher Scientific-Pierce. An amount of 30 μg t of protein for each sample was loaded and resolved onto a 10% Bolt Bis-Tris plus mini gel as previously described [35]. The iBlot® 2 gel transfer stacks mini integrated with nitrocellulose transfer membranes were used to transfer protein by using the dry blotting iBlot 2 gel transfer device (all provided by Thermo Fisher Scientific-Invitrogen, Waltham, MA, USA). The membranes were blocked in Tris-buffered saline (TBS) with 5% BSA (bovine serum albumin), then incubated with primary antibodies in 5% BSA, 0.1% Tween overnight at 4 °C and incubated with the appropriate secondary antibodies. Primary antibodies used were at a ratio of 1:1,000 were: anti-SR-B1 (Novus Biologicals, Littleton CO, USA) and anti-GAPDH (Cell Signaling Technology, Inc., Danvers, MA, USA), used as loading control. Detection was achieved by HRP-conjugated anti-rabbit (1:10000 dilution) (Jackson ImmunoResearch, Ely, Cambridgeshire, UK). Immunoreactive bands were detected by enhanced chemiluminescence using Clarity Western ECL Substrate (Bio-Rad Laboratories Inc., Hercules, CA, USA), and images were captured by iBright Imaging Systems (Thermo Fisher Scientific-Invitrogen, Waltham, MA, USA). Protein expression was quantified by densitometric analysis using ImageJ software (version 1.52a).

Cell immunofluorescence

siCTRL and silenced siSRB1 LX-2 and HepG2 cells were seeded at a density of 2 × 104 cells/well in a 4-well chamber slide (Nunc, Naperville, IL, USA) and incubated overnight in 1% FBS supplemented with DMEM at 37 °C and treated as described in Transfection section. After treatment, cells were fixed in 4% paraformaldehyde/PBS for 15 minutes at room temperature and washed three times with PBS. Cells were blocked with 5% bovine serum albumin in PBS for 1 hour followed by overnight incubation at 4°C with primary antibodies: rabbit polyclonal anti-SMAD2/3 antibody (Santa Cruz Biotechnology Inc., Dallas, TX, USA), and mouse monoclonal anti-PPARγ antibody (Santa Cruz Biotechnology Inc., Dallas, TX, USA). Then, after washing, samples were treated for 60 min at RT with a labeled isotype-specific secondary antibody, including 488AlexaFluor and 555AlexaFluor (Life Technologies-Invitrogen, Carlsbad, CA, USA). Cell nuclei were blue-stained with Hoechst 33342 (Life Technologies-Invitrogen, Carlsbad, CA, USA). The images of the immunostained cells were acquired by Evident-Olympus FV3000 (Evident Europe GmbH, Olympus, Microsystems, TOKYO, Japan) equipped with Fluoview (FV31S-SW). Representative images were captured and assembled using Adobe Photoshop CS6 software (Adobe Systems Inc., San Jose, CA, USA).

Supplementary Tables

Table S1. Probes for QRT-PCR in mice**.**

Gene name	**Mice**	**Human**
Glyceraldehyde-3-phosphate dehydrogenase (GAPDH)	Mm99999915_g1	Hs02786624_g1
Scavenger receptor class B type 1 (SRB1)	Mm00450234_m1	Hs00969821_m1
Fatty Acid Synthase (FAS)	Mm00662319_m1	Hs01005622_m1
Scavenger Receptor Class B, Member 3 (CD36)	Mm00432403_m1	Hs00354519_m1
Collagen Type I Alpha 1 Chain (COL1A1)	Mm.PT.56a.17374081	Hs00164004_m1
Collagen Type III Alpha 1 Chain (COL3A)	Mn00802300_m1	Hs00943809_m1
Smooth muscle alpha-actin (α-SMA)	Mm.PT.56a.30217320	Hs.00909449_m1
Transforming Growth Factor-β (TGFβ)	Hs.PT.49.1806981	Hs.PT.49.1806981
ATP Binding Cassette Subfamily A Member 1 (ABCA1)	Mm00442646_m1	-
ATP Binding Cassette Subfamily G Member 5 (ABCG5)	Mm00446241_m1	-
ATP binding cassette subfamily G member 8 (ABCG8)	Mm00445980_m1	-
Fatty Acid Binding Protein 4 (FABP4)	Mm00445878_m1	-
Carnitine palmitoyltransferase I (CPT)1	Mm01231183_m1	-
Stearoyl-CoA desaturase (SCID)	-	Hs01682761_m1

Table S2. Antibody array MAP.

	1	2	3	4	5	6	7	8	9	10	11	12
**a**	**POS**	**POS**	**NEG**	**NEG**	**BLANK**	**BLC**	**CD30 Ligand**	**Eotaxin-1**	**Eotaxin-2**	**Fas Ligand**	**Fractalkine**	**CCL11**
**b**	**POS**	**POS**	**NEG**	**NEG**	**BLANK**	**BLC**	**CD30 Ligand**	**Eotaxin-1**	**Eotaxin-2**	**Fas Ligand**	**Fractalkine**	**CCL11**
**c**	**GM-CSF**	**IFN-γ**	**IL-1α**	**IL-1β**	**IL-2**	**IL-3**	**IL-4**	**IL-6**	**IL-9**	**IL-10**	**IL-12**	**IL-12 p70**
**d**	**GM-CSF**	**IFN-γ**	**IL-1α**	**IL-1β**	**IL-2**	**IL-3**	**IL-4**	**IL-6**	**IL-9**	**IL-10**	**IL-12**	**IL-12 p70**
**e**	**IL-13**	**IL-17A**	**CXCL11**	**CXCL1**	**Leptin**	**LIX**	**XCL1**	**MCP-1**	**M-CSF**	**CXCL9**	**MIP-1α**	**MIP-1γ**
**f**	**IL-13**	**IL-17A**	**CXCL11**	**CXCL1**	**Leptin**	**LIX**	**XCL1**	**MCP-1**	**M-CSF**	**CXCL9**	**MIP-1α**	**MIP-1γ**
**g**	**RANTES**	**SDF-1α**	**I-309**	**CCL25**	**TIMP-1**	**TIMP-2**	**TNFα**	**TNF RI**	**TNF RII**	**BLANK**	**BLANK**	**NEG**
**h**	**RANTES**	**SDF-1α**	**I-309**	**CCL25**	**TIMP-1**	**TIMP-2**	**TNFα**	**TNF RI**	**TNF RII**	**BLANK**	**BLANK**	**NEG**

Table S3. TaqMan® probes for QRT-PCR Open Array.

5730403B10Rik_Mm00481784_m1	Ccr6_Mm99999114_s1	Fabp4_Mm00445878_m1	Il13ra1_Mm01302068_m1	Ltbp4_Mm00723639_g1	Serpina1a_Mm02748447_g1
A2m_Mm00558642_m1	Ccr7_Mm01301785_m1	Fabp4_Mm01295675_g1	Il13ra2_Mm00515166_m1	Ltbr_Mm00440235_m1	Serpina3c_Mm00434669_m1
Abcb1a_Mm00440761_m1	Ccr8_Mm00843415_s1	Fam3b_Mm00508056_m1	Il15_Mm00434210_m1	Ltf_Mm00434787_m1	Serpinf2_Mm00435868_m1
Abcf1_Mm01275245_m1	Ccr8_Mm99999115_s1	Fam3c_Mm00506835_m1	Il15ra_Mm00500457_m1	Ly75_Mm00522144_m1	Serping1_Mm00437834_m1
Actb_Mm00607939_s1	Ccr9_Mm02620030_s1	Fas_Mm00433237_m1	Il16_Mm00516039_m1	Ly86_Mm00440240_m1	Sftpa1_Mm00499170_m1
Acvr1_Mm01331069_m1	Ccrl1_Mm02620636_s1	Fasl_Mm00438864_m1	Il17a_Mm00439618_m1	Lyn_Mm01217488_m1	Sh2b2_Mm00517020_m1
Acvr2b_Mm00431664_m1	Ccrl2_Mm00516914_g1	Fcer1a_Mm00438867_m1	Il17c_Mm00521397_m1	Malt1_Mm00555961_m1	Siglec1_Mm00488332_m1
Acvrl1_Mm00437432_m1	Cd14_Mm00438094_g1	Fcer1g_Mm02343757_m1	Il17d_Mm01313472_m1	Map2k3_Mm00435950_m1	Siva1_Mm00834449_g1
Adipoq_Mm00456425_m1	Cd163_Mm00474091_m1	Fcgr2b_Mm00438875_m1	Il17f_Mm00521423_m1	Map2k6_Mm00803694_m1	Slco1a4_Mm00453126_m1
Adora1_Mm01308023_m1	Cd180_Mm00434804_m1	Fgf10_Mm00433275_m1	Il17ra_Mm00434214_m1	Mapk14_Mm00442497_m1	Slurp1_Mm00445117_m1
Adora2a_Mm00802075_m1	Cd1d1_Mm00783541_s1	Fgf11_Mm00679875_m1	Il17rb_Mm00444704_m1	Mapk8_Mm00489514_m1	Socs1_Mm00782550_s1
Adora3_Mm00802076_m1	Cd24a_Mm00782538_sH	Fgf12_Mm00802587_m1	Il18_Mm00434225_m1	Mapkapk2_Mm01288465_m1	Socs2_Mm00850544_g1
Adrb2_Mm02524224_s1	Cd274_Mm00452054_m1	Fgf18_Mm00433286_m1	Il18bp_Mm00456733_m1	Masp1_Mm00434830_m1	Sod1_Mm01344232_g1
Afap1l2_Mm00525039_m1	Cd28_Mm00483137_m1	Fgf20_Mm00748347_m1	Il18rap_Mm00516053_m1	Masp2_Mm00521963_m1	Sod1_Mm01700393_g1
Aif1_Mm00479862_g1	Cd40_Mm00441891_m1	Fgf23_Mm00445621_m1	Il19_Mm01288324_m1	Mbl2_Mm00487623_m1	Spp1_Mm00436767_m1
Aimp1_Mm00433034_m1	Cd40lg_Mm00441911_m1	Fgf2_Mm00433287_m1	Il1a_Mm00439620_m1	Mefv_Mm00490258_m1	Spred1_Mm00473782_m1
Aimp1_Mm01320868_m1	Cd44_Mm01277163_m1	Figf_Mm01131929_m1	Il1b_Mm01336189_m1	Mgll_Mm00449274_m1	Spred2_Mm00835803_g1
Akt1_Mm01331626_m1	Cd46_Mm00487625_m1	Flt3l_Mm00442801_m1	Il1b_Mm99999061_mH	Mmp25_Mm01309189_m1	Stab1_Mm00460390_m1
Alox15_Mm00507789_m1	Cd4_Mm00442754_m1	Fn1_Mm01256744_m1	Il1f10_Mm00462022_g1	Mrc1_Mm00485148_m1	Stat1_Mm00439531_m1
Alox5_Mm01182740_g1	Cd55_Mm00438377_m1	Fos_Mm00487425_m1	Il1f5_Mm00497802_m1	Mstn_Mm01254559_m1	Stat3_Mm01219775_m1
Alox5_Mm01182743_m1	Cd70_Mm00441914_m1	Fos_Mm00487426_g1	Il1f6_Mm00457645_m1	Muc1_Mm00449604_m1	Stat4_Mm00448890_m1
Alox5_Mm01182747_m1	Cd74_Mm00658576_m1	Foxp3_Mm00475162_m1	Il1f9_Mm00463327_m1	Muc4_Mm00466886_m1	Stat5b_Mm00839889_m1
Alox5_Mm01182748_m1	Cd80_Mm00711660_m1	Fpr1_Mm00442803_s1	Il1r1_Mm00434237_m1	Myd88_Mm00440338_m1	Stat6_Mm01160477_m1
Alox5_Mm01182749_m1	Cd86_Mm00444543_m1	Fpr3_Mm01962454_s1	Il1rap_Mm00492638_m1	Ncam1_Mm01149710_m1	Tacr1_Mm00436892_m1
Alox5_Mm01182750_m1	Cd97_Mm00516248_m1	Gal_Mm00439056_m1	Il1rapl2_Mm00472725_m1	Ncf1_Mm00447921_m1	Tbp_Mm00446973_m1
Alox5ap_Mm00802100_m1	Cdk5_Mm01164910_m1	Gapdh_Mm99999915_g1	Il1rl1_Mm00516117_m1	Ndst1_Mm00447005_m1	Tfrc_Mm00441941_m1
Anxa1_Mm00440225_m1	Cdkn1a_Mm00432448_m1	Gdf11_Mm01159973_m1	Il1rl2_Mm00519250_m1	Nfam1_Mm00546934_m1	Tgfb1_Mm01178820_m1
Aoah_Mm00600104_m1	Cdo1_Mm00473573_m1	Gdf15_Mm00442228_m1	Il1rn_Mm01337566_m1	Nfatc3_Mm01249200_m1	Tgfb2_Mm00436955_m1
Aoc3_Mm00839624_m1	Cebpa_Mm00514283_s1	Gdf2_Mm00807340_m1	Il20_Mm00445341_m1	Nfatc4_Mm01323917_m1	Tgfbr1_Mm00436964_m1
Aox1_Mm00437475_m1	Cebpb_Mm00843434_s1	Gdf3_Mm00433563_m1	Il21_Mm00517640_m1	Nfe2l1_Mm00599712_m1	Tgfbr1_Mm03024015_m1
Apcs_Mm00488099_g1	Cer1_Mm00515474_m1	Gdf5_Mm00433564_m1	Il22	Nfkb1_Mm00476361_m1	Tgm2_Mm00436987_m1
Apoa1_Mm00437568_g1	Cfb_Mm00433909_m1	Gdf6_Mm01222341_m1	Il22ra1_Mm00663697_m1	Nfrkb_Mm00555264_m1	Thpo_Mm00437040_m1
Apoa1_Mm00437569_m1	Cfd_Mm00442664_m1	Gdf9_Mm00433565_m1	Il22ra2_Mm00617572_m1	Nfx1_Mm00458401_m1	Timm50_Mm00508510_m1
Apoa4_Mm00431814_m1	Cfh_Mm01299243_m1	Gh_Mm00433590_g1	Il23a_Mm00518984_m1	Nlrc4_Mm01233151_m1	Tirap_Mm00446502_m1
Apoe_Mm00437573_m1	Cfhr1_Mm00502018_m1	Ghrl_Mm00445450_m1	Il23a_Mm01160011_g1	Nlrp3_Mm00840904_m1	Tlr11_Mm01701924_s1
Areg_Mm00437583_m1	Cfi_Mm00432470_m1	Ghsr_Mm00616415_m1	Il23r_Mm00519943_m1	Nmi_Mm00803857_m1	Tlr12_Mm01180204_s1
Atrn_Mm00437746_m1	Cfp_Mm01341415_m1	Glmn_Mm00504709_m1	Il24_Mm00474102_m1	Nod1_Mm00805062_m1	Tlr13_Mm01233819_m1
Axl_Mm00437221_m1	Cftr_Mm00445197_m1	Gpr17_Mm02619401_s1	Il27_Mm00461164_m1	Nod2_Mm00467543_m1	Tlr1
B2m_Mm00437762_m1	Chi3l3_Mm00657889_mH	Gpr68_Mm00558545_s1	Il27ra_Mm00497259_m1	Nodal_Mm00443040_m1	Tlr1_Mm00446095_m1

B4galt1_Mm00480752_m1	Chrna7_Mm01312230_m1	Gpx1_Mm00656767_g1	Il28b_Mm00663660_g1	Nono_Mm00834875_g1	Tlr1_Mm01208874_m1
Bad_Mm00432042_m1	Chst1_Mm00517855_m1	Gpx4_Mm00515041_m1	Il28ra_Mm00558035_m1	Nos2_Mm00440502_m1	Tlr2_Mm00442346_m1
Bcl10_Mm00784755_s1	Chst2_Mm00490018_g1	Grem1_Mm00488615_s1	Il28ra_Mm01192973_m1	Nox4_Mm00479246_m1	Tlr3_Mm00628112_m1
Bcl6_Mm00477633_m1	Chst4_Mm00488783_s1	Grem2_Mm00501909_m1	Il2_Mm00434256_m1	Nr3c1_Mm00433832_m1	Tlr4_Mm00445273_m1
Bdkrb1_Mm04207315_s1	Cklf_Mm00459364_m1	Grn_Mm00433848_m1	Il2_Mm99999222_m1	Nup85_Mm01243354_m1	Tlr5_Mm00546288_s1
Bdkrb2_Mm00437788_s1	Clcf1_Mm00480200_m1	Gsk3b_Mm00444911_m1	Il2rg_Mm00442885_m1	Oit1_Mm00455341_m1	Tlr6_Mm02529782_s1
Blnk_Mm01197846_m1	Clec7a_Mm01183349_m1	Gusb_Mm00446953_m1	Il31ra_Mm00519844_m1	Olr1_Mm00454586_m1	Tlr7_Mm00446590_m1
Bmp10_Mm01183889_m1	Clu_Mm00442773_m1	Gusb_Mm01197698_m1	Il33_Mm00505403_m1	Orm1_Mm00435456_g1	Tlr9_Mm00446193_m1
Bmp15_Mm00437797_m1	Cmklr1_Mm01700212_m1	Gusb_Mm03003537_s1	Il3_Mm00439631_m1	Osm_Mm01193966_m1	Tnc_Mm00495662_m1
Bmp1_Mm00802225_m1	Cmtm2a_Mm00459052_m1	H2-Q10_Mm01275264_g1	Il3ra_Mm00434273_m1	Oxgr1_Mm01960674_s1	Tnf_Mm00443258_m1
Bmp2_Mm01340178_m1	Cmtm2b_Mm00459292_m1	H47_Mm00502826_m1	Il4_Mm00445259_m1	P2rx1_Mm00435460_m1	Tnfaip3_Mm00437121_m1
Bmp3_Mm00557790_m1	Cmtm3_Mm00470315_m1	Havcr2_Mm00454540_m1	Il4ra_Mm00439634_m1	P2rx7_Mm00440578_m1	Tnfaip6_Mm00493736_m1
Bmp3_Mm03024297_s1	Cmtm4_Mm00463816_m1	Hc_Mm00439275_m1	Il5_Mm00439646_m1	P2ry1_Mm00435471_m1	Tnfrsf11b_Mm01205928_m1
Bmp4_Mm00432087_m1	Cmtm5_Mm00509113_m1	Hdac4_Mm01299543_m1	Il5ra_Mm00434284_m1	Pdgfb_Mm00440677_m1	Tnfrsf14_Mm00619239_m1
Bmp5_Mm00432091_m1	Cmtm6_Mm00509048_m1	Hdac4_Mm01299557_m1	Il6_Mm00446190_m1	Pgk1_Mm00435617_m1	Tnfrsf18_Mm00437136_m1
Bmp6_Mm00432095_m1	Cmtm7_Mm00506011_m1	Hdac4_Mm01299565_m1	Il6_Mm01210732_g1	Pglyrp1_Mm00437150_m1	Tnfrsf19_Mm00443506_m1
Bmp6_Mm01332882_m1	Cmtm8_Mm00510311_m1	Hdac5_Mm00515917_m1	Il6_Mm01210733_m1	Pik3r1_Mm00803160_m1	Tnfrsf1a_Mm00441875_m1
Bmp7_Mm00432101_m1	Cntfr_Mm00516693_m1	Hdac5_Mm00515941_g1	Il6ra_Mm00439653_m1	Pla2g2d_Mm00478250_m1	Tnfrsf1a_Mm01182929_m1
Bmp7_Mm00432102_m1	Cntfr_Mm00516697_m1	Hdac5_Mm01246076_m1	Il6st_Mm00439665_m1	Pla2g2e_Mm00478870_m1	Tnfrsf1b_Mm00441889_m1
Bmp7_Mm00432105_m1	Cntnap1_Mm00489702_m1	Hdac7_Mm00469520_m1	Il7r_Mm00434295_m1	Pla2g4c_Mm01195718_m1	Tnfrsf25_Mm01263821_m1
Bmp8a_Mm00432109_m1	Cr1l_Mm00785297_s1	Hdac7_Mm00469527_m1	Il8ra_Mm00731329_s1	Pla2g7_Mm00479105_m1	Tnfrsf4_Mm00442039_m1
Bmp8b_Mm00432115_g1	Cr2_Mm00801681_m1	Hdac9_Mm01293999_m1	Il8rb_Mm00438258_m1	Plaa_Mm00554584_m1	Tnfrsf8_Mm00437140_m1
Bmpr1b_Mm00432117_m1	Crh_Mm01293920_s1	Hgf_Mm01135182_m1	Il9_Mm00434305_m1	Plp2_Mm02342686_g1	Tnfrsf9_Mm00441899_m1
Bre_Mm00513816_m1	Crlf1_Mm00517026_m1	Hgf_Mm01135183_m1	Inha_Mm00439683_m1	Polr2a_Mm00839493_m1	Tnfsf10_Mm01283606_m1
C1qa_Mm00432142_m1	Crp_Mm00432680_g1	Hgf_Mm01135193_m1	Inhba_Mm00434339_m1	Pparg_Mm01184322_m1	Tnfsf11_Mm00441906_m1
C1qb_Mm01179619_m1	Csf1_Mm00432686_m1	Hif1a_Mm01283760_m1	Inhbb_Mm01286587_m1	Pparg_Mm01184323_m1	Tnfsf12
C1qc_Mm01193990_g1	Csf2_Mm00438328_m1	Hmbs_Mm00660262_g1	Ins2_Mm00731595_gH	Ppbp_Mm00470163_m1	Tnfsf13
C1rl_Mm00619332_m1	Csf2_Mm01290062_m1	Hmgb1_Mm00849805_gH	Ipo8_Mm01255158_m1	Ppia	Tnfsf13b_Mm00446347_m1
C1s_Mm00663210_mH	Csf2ra_Mm00438331_g1	Hmox1_Mm00516005_m1	Irak1_Mm00434254_m1	Ppia_Mm02342429_g1	Tnfsf14_Mm00444567_m1
C2_Mm00442726_m1	Csf2rb_Mm00655745_m1	Hprt1_Mm00446968_m1	Irak2_Mm00549143_m1	Ppia_Mm02342430_g1	Tnfsf15_Mm00770031_m1
C3_Mm00437858_m1	Csf3_Mm00438334_m1	Hprt1_Mm01318743_m1	Irak3_Mm00518541_m1	Prdx5_Mm00465365_m1	Tnfsf18_Mm00839222_m1
C3ar1_Mm01184110_m1	Csf3_Mm00438335_g1	Hprt1_Mm01318747_g1	Irak4_Mm00459443_m1	Prg2_Mm00435905_m1	Tnfsf4_Mm00437214_m1
C3ar1_Mm02620006_s1	Ctf1_Mm00432772_m1	Hprt1_Mm01324427_m1	Irf3_Mm00516779_m1	Prl7d1_Mm00599916_m1	Tnfsf8_Mm00437153_m1
C6_Mm00489521_m1	Ctla4_Mm00486849_m1	Hpse_Mm00461768_m1	Irf3_Mm01203177_m1	Procr_Mm00440992_m1	Tnfsf9_Mm00437155_m1
C8a_Mm00521625_m1	Cx3cl1_Mm00436454_m1	Hrh1_Mm00434002_s1	Irf7_Mm00516788_m1	Prok2_Mm00450080_m1	Tollip_Mm00445841_m1
C8b_Mm00804806_m1	Cx3cr1_Mm02620111_s1	Hrh1_Mm00627039_m1	Itgal_Mm00801807_m1	Prok2_Mm01182451_m1	Tpst1_Mm00496931_m1
C8g_Mm01330461_g1	Cxcl10_Mm00445235_m1	Hsp90ab1_Mm00833431_g1	Itgam_Mm00434455_m1	Prtn3_Mm00478323_m1	Trem1_Mm00451738_m1
C9_Mm00442739_m1	Cxcl11_Mm00444662_m1	Hspd1_Mm00849835_g1	Itgb2_Mm00434513_m1	Ptafr_Mm02621061_m1	Trf_Mm00446708_m1
Camp_Mm00438285_m1	Cxcl12_Mm00445553_m1	Icos_Mm00497600_m1	Itgb2l_Mm00492710_m1	Pten_Mm00477208_m1	Trip6_Mm00600041_m1
Casp1_Mm00438023_m1	Cxcl14_Mm00444699_m1	Icosl_Mm00497237_m1	Itgb6_Mm01269869_m1	Ptges_Mm00452105_m1	Trp53_Mm01731287_m1
Casp8_Mm00802247_m1	Cxcl16_Mm00469712_m1	Ifih1_Mm00459183_m1	Jak1_Mm00600614_m1	Ptgs1_Mm00477214_m1	Trpv1_Mm01246302_m1
Cav1_Mm00483057_m1	Cxcl17_Mm00463791_m1	Ifna12_Mm00616656_s1	Jak2_Mm01208489_m1	Ptgs2_Mm00478374_m1	Tslp_Mm00498739_m1

Ccbp2_Mm00445551_m1	Cxcl1_Mm00433859_m1	Ifna14_Mm01703465_s1	Jak3_Mm00439962_m1	Ptn_Mm00436062_m1	Twist1_Mm00442036_m1
Ccl11_Mm00441238_m1	Cxcl1_Mm04207460_m1	Ifna2_Mm00833961_s1	Jun_Mm00495062_s1	Ptpn6_Mm00469153_m1	Txlna_Mm01185793_m1
Ccl17_Mm00516136_m1	Cxcl2_Mm00436450_m1	Ifna4_Mm00833969_s1	Kit_Mm00445212_m1	Ptprc_Mm01293575_m1	Ubc_Mm01201237_m1
Ccl19_Mm00839967_g1	Cxcl3_Mm01701838_m1	Ifnar1_Mm00439544_m1	Kitl_Mm00442972_m1	Reg3g_Mm00441127_m1	Unc13d_Mm01252606_m1
Ccl1_Mm00441236_m1	Cxcl5_Mm00436451_g1	Ifnar2_Mm00494916_m1	Klf6_Mm00516184_m1	Rela_Mm00501346_m1	Vegfa_Mm01281449_m1
Ccl20_Mm01268754_m1	Cxcl9_Mm00434946_m1	Ifnb1_Mm00439552_s1	Klkb1_Mm00434658_m1	Retn_Mm00445641_m1	Vegfb_Mm00442102_m1
Ccl22_Mm00436439_m1	Cxcr3_Mm00438259_m1	Ifne_Mm00616542_s1	Klrg1_Mm00516879_m1	Rhoa_Mm00834507_g1	Vegfc_Mm00437310_m1
Ccl24_Mm00444701_m1	Cxcr3_Mm99999054_s1	Ifng_Mm01168134_m1	Kng1_Mm00445679_m1	Rhoa_Mm01228062_g1	Vip_Mm00660234_m1
Ccl25_Mm00436443_m1	Cxcr4_Mm01292123_m1	Ifng_Mm99999071_m1	Krt1_Mm00492992_g1	Rhoa_Mm01601614_g1	Vps45_Mm00496940_m1
Ccl26_Mm02763057_u1	Cxcr6_Mm02620517_s1	Ifngr1_Mm00599890_m1	Krt7_Mm00466676_m1	Ripk2_Mm00446816_m1	Wnt16_Mm00446420_m1
Ccl28_Mm00445039_m1	Cxcr7_Mm02619632_s1	Ifngr2_Mm00492626_m1	Krt8_Mm00835759_m1	Rplp2_Mm00782638_s1	Xcl1_Mm00434772_m1
Ccl2_Mm00441242_m1	Cybb_Mm01287743_m1	Ifnk_Mm02529417_s1	Lbp_Mm00493139_m1	Ryr1_Mm01175211_m1	Xcr1_Mm00442206_s1
Ccl3_Mm00441258_m1	Cyp26b1_Mm00558507_m1	Igf1_Mm00439560_m1	Lefty1	S100a8_Mm00496696_g1	Ywhaz_Mm01158417_g1
Ccl4_Mm00443111_m1	Darc_Mm04207950_g1	Igfbp4_Mm00494922_m1	Lefty2_Mm00774547_m1	S100a8_Mm01220132_g1	Zfp36_Mm00457144_m1
Ccl5_Mm01302427_m1	Ddx58_Mm00554529_m1	Ik_Mm00803668_m1	Lefty2_Mm03024313_s1	S100a9_Mm00656925_m1	
Ccl5_Mm01302428_m1	Dnajc8_Mm00552449_m1	Il10_Mm00439614_m1	Lefty2_Mm03053439_g1	S100b_Mm00485897_m1	
Ccl6_Mm01302419_m1	Ebi3_Mm00469294_m1	Il10ra_Mm00434151_m1	Lep_Mm00434759_m1	S1pr1_Mm02619656_s1	
Ccl7_Mm00443113_m1	Eda2r_Mm00723601_m1	Il10rb_Mm00434157_m1	Lepr_Mm00440181_m1	S1pr3_Mm04229896_m1	
Ccl7_Mm01308393_g1	Egfr_Mm00433023_m1	Il12a_Mm00434165_m1	Lif_Mm00434762_g1	Saa1_Mm00656927_g1	
Ccl8_Mm01297183_m1	Egfr_Mm01187858_m1	Il12a_Mm99999066_m1	Lifr_Mm00442940_m1	Saa3_Mm00441203_m1	
Ccr1_Mm00438260_s1	Ela2_Mm00469310_m1	Il12b_Mm00434174_m1	Lifr_Mm00442942_m1	Scgb3a1_Mm00468033_g1	
Ccr1l1_Mm00432606_s1	Ereg_Mm00514794_m1	Il12b_Mm99999067_m1	Lilrb3_Mm01700366_m1	Scn9a_Mm00450762_s1	
Ccr2_Mm00438270_m1	F11r_Mm00554113_m1	Il12rb1_Mm00434189_m1	Lrp8_Mm00474028_m1	Scube1_Mm00491651_m1	
Ccr3_Mm00515543_s1	F2_Mm00438843_m1	Il12rb2_Mm00434200_m1	Lta4h_Mm00521826_m1	Sdcbp_Mm00489742_m1	
Ccr3_Mm01216172_m1	F2r_Mm00438851_m1	Il13_Mm00434204_m1	Ltb4r1_Mm00521839_m1	Sectm1b_Mm00459026_m1	
Ccr4_Mm00438271_m1	F2rl1_Mm00433160_m1	Il13_Mm99999190_m1	Ltb_Mm00434774_g1	Sele_Mm00441278_m1	
Ccr5_Mm01216171_m1	F3_Mm00438853_m1	Il13ra1_Mm00446726_m1	Ltbp4_Mm00723631_m1	Selp_Mm00441295_m1	

Table S4. Expression data of inflammatory genes dysregulated genes in WD/CCl4 mice with or without intestinal activation of LXRα. Mean Relative quantification (RQ) the expression of 382 dysregulated genes involved in inflammatory response that were included in a TaqMan OpenArray Mouse Inflammation Panel. The relative expression was assessed in WT+WD/CCl4, iLXRα+ND, iLXRα+WD/CCl4 respect to WT+ND samples.

Gene name	WT+ND (mean RQ)	WT+WD/CCl4 (mean RQ)	iLXRα+ND (mean RQ)	iLXRα+WD/CCl4 (mean RQ)
573Rik	1.444	0.6535	1.4315	0.859
Abcf1	1.227	0.71	1.0595	0.8475
Actb	1.225	1.9055	1.7695	1.234
Acvr1	1.473	1.0675	1.703	0.974
Acvr2b	0.938	0.325	0.571	0.3
Acvrl1	0.9065	1.9725	1.324	0.01
Adrb2	1.5625	3.4815	1.395	4.437
Afap1l2	1.271	1.4285	2.771	3.0815
Aif1	0.969	3.399	1.2695	2.4075
Aimp1	1.096	0.631	1.0385	0.8745
Aimp1	1.3365	0.897	1.3665	1.2255
Alox5	1.4115	1.3255	0	6.5045
Alox5ap	1.124	2.32	1.2135	2.9585
Anxa1	1.6765	10.0485	1.992	6.8515
Aoah	1.068	3.964	1.214	4.227
Aox1	1.203	0.524	1.6175	0.799
Apcs	1.717	1.186	1.845	1.8215
Apoa1	1.514	0.8195	1.5135	0.8825
Apoa1	1.4545	0.7305	1.548	0.844
Apoa4	1.881	12.9485	1.557	14.7285
Apoe	1.275	0.976	1.275	1.006
Atrn	1.544	1.302	1.797	0.9355
Axl	1.3695	3.404	1.6235	2.717
B2m	1.2815	1.0455	1.392	1.1375
Bad	1.065	0.745	1.1885	0.803
Bcl10	1.314	1.2795	1.743	1.282
Bdkrb2	1.2415	0	3.1185	0.482
Blnk	0.82	5.515	0.614	2.3075
Bmp10	1.0915	0.5105	1.3445	0.8675
Bmp1	1.5005	0.712	1.575	0.9975
Bmp2	1.6505	0.9075	1.185	0.787
Bmp4	1.2495	1.131	1.3655	0.983
Bmp5	1.18	1.5685	1.6985	1.387
Bmp6	1.2285	0.496	1.3495	0.587
Bmp6	1.4155	0.38	1.405	0.744
Bmp7	1.049	0.6435	3.166	0.614

Bmp7	0.705	0.2215	2.003	0.2745
Bmp7	0.8395	0.4105	4.03	0.8715
Bmp8b	0.8065	1.1015	0	1.851
Bre	1.348	0.961	1.3355	1.2885
C1qa	1.0855	2.87	1.3	2.327
C1qb	1.2915	3.799	1.2385	3.19
C1qc	1.177	4.8595	1.4365	3.5485
C1rl	1.241	0.582	1.319	0.837
C1s	1.224	0.815	1.4385	0.9105
C2	1.12	0.851	1.269	1.0655
C3	1.319	0.9025	1.566	1.015
C3ar1	1.07	11.7305	1.694	8.9135
C3ar1	1.5005	12.0905	1.4545	6.214
C6	1.6735	1.012	1.8755	1.5125
C8a	1.0705	0.524	1.0745	0.8635
C8b	1.1825	0.435	1.426	0.6605
C8g	1.189	0.7	1.282	0.742
C9	1.2225	0.822	1.2705	0.9805
Casp1	1.3915	4.3965	2.7525	3.486
Casp8	1.226	0.9515	1.6885	0.9235
Ccbp2	11.0815	3.8635	9.0735	7.701
Ccl19	1.074	4.7765	1.7815	3.268
Ccl22	0.5	13.44	0.8115	6.4945
Ccl25	1.288	0.584	1.133	0.5745
Ccl28	1.4325	0.6275	0.5605	0.928
Ccl2	2.1295	37.713	2.52	19.189
Ccl4	0.903	10.5515	1.168	8.895
Ccl5	1.075	4.3755	1.72	2.971
Ccl5	0.9585	3.2895	1.2255	1.729
Ccl6	1.575	9.3065	1.653	10.114
Ccr1	2.696	14.238	3.4325	22.891
Ccr2	1.582	12.0525	2.1675	9.153
Ccr3	1.162	1.8485	1.4725	2.142
Ccr3	1.3315	3.4245	2.877	2.876
Ccr5	1.177	4.484	1.2615	2.963
Ccr9	1.5095	1.27	1.627	0.947
Ccrl1	1.2535	0.559	1.311	0.8105
Ccrl2	1.7115	3.632	1.6195	2.766
Cd14	1.5615	7.369	0.9025	5.3765
Cd163	1.0255	0.02	0.7845	0.9195
Cd180	0.7865	10.699	1.759	3.804
Cd1d1	1.456	0.548	1.561	0.956

Cd24a	1.337	2.047	2.9915	3.14
Cd274	1.013	4.6775	1.991	2.6155
Cd28	0.5	1.8615	0.8265	1.4425
Cd40	0.8825	3.9615	1.3505	3.1715
Cd44	1.248	6.3975	1.9115	4.8775
Cd74	1.0975	3.892	1.066	2.092
Cd86	1.7265	6.4425	2.9035	4.0115
Cd97	1.2635	2.292	1.663	2.18
Cdk5	1.091	0.57	0.9055	0.637
Cdkn1a	2.3115	33.9575	1.885	20.8945
Cdo1	1.6705	1.401	1.87	1.036
Cebpa	1.292	1.2885	1.976	0.8195
Cebpb	0.7725	0.9575	0.6965	0.1655
Cfb	1.052	0.779	1.147	0.869
Cfh	1.217	0.923	1.274	1.033
Cfhr1	1.152	0.589	1.8425	0.6655
Cfp	0.946	1.5885	0.6595	1.5375
Chst2	0.9455	1.44	1.887	0.1575
Cklf	1.141	2.442	3.164	2.0935
Clcf1	2.4015	5.3015	2.8075	1.8915
Clec7a	0.6255	25.7075	1.214	11.792
Clu	1.293	0.9995	1.233	1.05
Cmklr1	1.0415	1.755	1.218	1.966
Cmtm3	1.1595	2.36	1.638	2.1745
Cmtm4	1.006	0.4495	0.9335	0.2905
Cmtm5	0.9695	0	0.4245	0.2575
Cmtm6	1.3835	1.037	1.876	1.077
Cmtm7	1.692	4.971	3.293	5.2315
Cmtm8	1.1385	0.5725	1.1185	0.757
Cntnap1	1.9595	6.878	4.1355	19.469
Cr1l	1.2045	1.059	1.4365	1.0555
Cr2	0.505	4.882	0.534	2.412
Csf1	1.022	2.7195	1.639	2.6995
Csf2	1.3145	1.506	0	0
Csf2ra	0.947	4.099	1.0725	1.9185
Csf2rb	1.531	9.695	1.4835	6.2935
Ctf1	1.251	1.2395	1.391	1.002
Cx3cl1	3.3565	15.8825	10.0245	14.778
Cx3cr1	7.8845	58.3765	10.7165	39.5205
Cxcl10	0.728	7.156	1.9005	5.3625
Cxcl12	1.3345	0.7015	1.6395	0.86
Cxcl14	0.767	36.1715	11.8185	10.883

Cxcl16	1.2335	3.706	1.669	2.9805
Cxcl2	0.893	8.3125	0.381	8.773
Cxcl9	0.988	4.29	0.874	1.8235
Cxcr3	1.0565	1.7625	1.2745	1.6895
Cxcr3	1.6455	2.839	0.86	3.109
Cxcr4	1.376	6.97	4.502	7.1715
Cybb	1.2295	7.831	1.361	4.1275
Cyp26b1	1.134	0.3465	1.029	0.4825
Darc	1.0195	1.5955	1.7215	1.207
Ddx58	1.1465	1.665	1.4645	2.252
Dnajc8	1.4505	1.1455	1.604	1.19
Ebi3	2.4825	3.395	1.547	2.8665
Eda2r	0.6745	17.065	1.8485	12.3805
Egfr	1.1225	0.319	0.7705	1.0225
Egfr	1.246	0.4085	0.828	1.0965
Ephx2	1.4685	0.488	1.237	0.676
Erap1	1.1885	0.8355	1.252	1.0135
Ereg	1.832	3.554	0.9185	4.2125
F11r	1.196	0.7345	1.275	0.859
F2	1.186	0.5605	1.126	0.6075
F2r	1.125	1.779	1.499	2.3115
F2rl1	0.5	4.886	1.74	5.938
F3	0.995	0.867	1.106	0.7855
Fabp4	1.93	4.699	14.4185	7.2985
Fas	1.4315	1.975	2.288	2.0355
Fasl	3.5075	0.5185	0.9325	2.153
Fcer1g	1.1245	6.627	1.579	3.9775
Fgf12	1.3505	0.9415	1.8565	0.6175
Flt3l	1.1675	1.929	1.1045	1.6075
Fn1	1.4715	0.635	1.3635	0.9955
Fos	0.674	0.203	0.8235	0.13
Fos	0.749	0.1445	0.758	0.0885
Fpr1	1.2	2.62	2.2985	4.357
Gapdh	1.33	1.2995	1.563	1.3
Gdf15	0.9625	0.5615	0.726	0.837
Gdf2	1.0025	1.239	0.994	1.2075
Gdf6	2.765	7.3915	0	8.548
Gdf9	0.811	0	1.674	0
Glmn	1.095	1.4675	1.4335	1.3805
Gpr17	2.0205	0.488	1.3365	1.297
Gpr68	2.295	3.166	2.151	2.0215
Gpx1	1.2895	0.9275	1.362	0.9735

Gpx4	1.1805	0.8535	1.245	0.947
Gusb	1.0945	2.21	1.206	1.7625
Gusb	0.985	1.735	0.9215	1.316
Gusb	1.06	1.816	1.0555	1.3295
H2-Q10	1.245	0.687	1.233	0.7745
H47	1.4095	0.8855	1.624	0.874
Havcr2	1.2305	9.5245	1.825	7.2205
Hc	1.4655	0.721	1.55	1.077
Hdac4	1.054	0.5455	0.9965	0.5905
Hdac4	1.048	0.7305	1.2265	0.869
Hdac5	1.0665	0.8375	1.275	0.8025
Hdac7	1.754	2.0055	2.9745	2.564
Hdac7	1.059	1.042	1.4295	1.5565
Hdac9	1.1555	2.2925	1.566	1.9605
Hgf	1.4645	1.982	1.8845	1.6905
Hgf	1.5095	2.062	1.8675	2.0345
Hif1a	1.121	0.854	1.212	0.78
Hmgb1	1.2545	0.8215	1.2985	0.8415
Hmox1	1.0795	1.2285	1.137	1.3515
Hprt1	1.4425	1.0915	1.8165	1.3355
Hprt1	1.2835	1.0605	1.503	1.0955
Hprt1	1.554	1.173	1.758	1.4785
Hprt1	1.431	1.167	1.8095	1.4215
Hpse	1.529	2.704	1.787	2.971
Hsp90ab1	1.2715	0.6215	0.992	0.853
Hspd1	1.3965	0.9065	1.375	0.9545
Icosl	0.663	3.0385	3.2365	2.323
Ifih1	1.3515	2.097	1.5815	3.173
Ifnar1	1.4345	1.1045	1.5125	0.958
Ifnar2	1.474	0.7895	1.4855	0.8285
Ifngr1	1.3775	2.185	1.491	1.7995
Ifngr2	1.39	1.854	1.617	1.317
Ifnk	1.629	0.772	1.5415	0.8705
Igf1	1.2265	0.6465	1.3065	0.9345
Igfbp4	1.2905	0.56	1.2875	0.706
Il10	1.008	0.593	0	0.483
Il10ra	0.8865	3.2355	1.6615	3.053
Il10rb	1.319	3.378	1.634	2.5105
Il12rb1	1.267	0.1455	0.85	0.35
Il12rb2	1.569	1.438	2.338	0.877
Il13ra1	1.516	1.687	1.524	1.89
Il13ra2	1.053	1.776	2.119	3.3495

Il15	0.8845	2.2565	1.58	1.32
Il15ra	1.1055	0.5625	1.006	0.65
Il17ra	1.193	0.885	1.2785	0.931
Il17rb	1.237	0.611	1.3685	0.8285
Il18	1.3175	0.9565	1.663	0.9515
Il18bp	1.054	2.849	1.0785	1.689
Il18rap	0.5	3.2605	3.403	2.28
Il1a	1.8875	1.9125	0.962	1.6685
Il1b	0.8885	1.431	0.791	0.371
Il1f9	0.5	8.462	0	9.041
Il1r1	1.0085	0.767	0.611	1.485
Il1rap	1.3575	0.515	1.433	0.659
Il1rn	2.576	13.278	2.89	9.1005
Il22ra1	0.9085	1.356	0.8825	1.43
Il23a	0.5	1.9995	2.082	1.062
Il23a	1.223	0.9295	0	1.024
Il27ra	0.9525	1.0415	0.585	1.017
Il2rg	0.8555	3.3275	1.433	16.0205
Il33	1.0465	2.6	1.574	5.274
Il3ra	1.1305	0.537	0.704	0.736
Il4ra	1.471	1.0815	1.8015	1.2915
Il6	1.2	3.1	1.43	0.49
Il6ra	1.1075	0.41	0.8045	0.5965
Il6st	1.368	1.033	1.653	1.208
Il7r	1.156	13.391	0.97	6.2805
Il8rb	0.5	4.831	1.9865	9.5745
Ipo8	1.1035	0.74	1.2875	0.833
Irak1	1.3265	0.862	1.431	1.178
Irak2	1.507	0.673	1.536	0.736
Irak3	1.074	8.541	2.397	3.663
Irak4	1.295	1.4325	1.283	1.57
Irf3	1.0745	0.7565	1.4495	1.249
Irf3	1.2185	0.809	1.465	0.951
Irf7	0.9225	1.627	0.848	2.343
Itgal	1.397	4.4045	1.9535	3.3675
Itgam	1.215	9.9845	0.97	10.966
Itgb2	0.9235	7.1135	1.479	5.147
Jak1	1.379	1.1145	1.5905	1.2275
Jak2	1.0695	1.0065	1.197	0.8585
Jak3	1.4175	2.3985	2.1865	2.841
Jun	0.93	0.4855	1.3895	0.5045
Klf6	1.1155	2.3025	1.337	2.033

Klkb1	1.397	0.5525	1.4965	0.6195
Kng1	1.147	0.4795	1.0405	0.5575
Krt7	0.81	1.5165	1.8155	2.3425
Krt8	1.4295	3.376	1.8685	2.6075
Lepr	1.0865	1.9145	0.844	3.386
Lifr	1.0665	0.4165	1.052	0.519
Lifr	1.355	0.5335	1.6435	0.778
Lilrb3	0.825	1.942	1.0945	1.849
Lta4h	1.3225	1.209	1.596	1.158
Ltb	1.0615	4.297	2.335	2.703
Ltbr	1.1445	0.8835	1.0415	0.9385
Ly75	1.061	1.3245	0.6705	0.957
Ly86	1.067	8.868	1.892	5.516
Map2k3	1.034	0.5945	0.833	0.699
Map2k6	1.2015	0.5785	0.796	0.626
Mapk14	1.1655	0.753	1.13	0.846
Mapk8	1.232	0.6765	1.2275	0.794
Mapkapk2	1.229	1.1285	1.272	0.919
Masp1	1.2195	0.7285	1.319	0.947
Masp2	1.341	0.8035	1.426	0.9495
Mbl2	1.289	0.662	1.2345	0.6235
Mefv	1.2435	3.5865	2.3505	4.8845
Mgll	1.2725	0.682	1.587	1.1735
Myd88	1.286	1.2205	1.4535	0.9125
Ncam1	0.5	17.727	41.247	27.6575
Ncf1	0.84	2.576	0.828	3.7975
Ndst1	1.2435	0.8495	1.6085	0.7885
Nfam1	0.904	3.728	1.3625	3.734
Nfatc3	1.26	0.86	1.4795	0.828
Nfe2l1	1.439	0.8325	1.7135	0.8815
Nfkb1	1.33	1.499	1.7515	1.4045
Nfrkb	1.488	1.116	1.7145	1.1575
Nfx1	1.1235	0.726	1.1635	0.731
Nlrc4	1.4185	9.542	3.106	12.2015
Nlrp3	0.8805	4.1335	2.339	3.102
Nono	1.146	0.657	1.208	1.0555
Nox4	1.3685	0.511	1.3425	0.6095
Nr3c1	1.2485	0.826	1.349	0.8655
Orm1	1.2565	1.0135	1.546	1.3745
P2rx7	1.8295	5.141	1.8345	3.717
P2ry1	1.4475	0.762	1.64	0.9615
Pdgfb	1.2125	8.3255	4.5145	6.581

Pgk1	1.212	0.8395	1.256	1.019
Pglyrp1	0.5	3.657	1.103	5.1775
Pik3r1	1.216	1.335	1.4545	1.4
Pla2g7	1.617	3.531	1.48	3.4405
Plaa	1.259	0.732	1.4605	0.9645
Polr2a	1.3185	1.018	1.6885	1.0445
Pparg	1.2045	3.5315	1.415	2.8705
Pparg	1.9325	3.8155	2.277	3.7015
Ppbp	1.2615	1.1165	2.8235	1.7015
Ppia	1.4625	1.0995	1.544	1.047
Ppia	1.262	0.8935	1.3315	0.841
Prdx5	1.221	0.723	1.1955	0.8655
Ptafr	2.977	74.922	2.8015	41.218
Pten	1.4705	0.8505	1.4555	0.967
Ptgs1	1.331	1.983	1.7805	1.964
Ptpn6	1.3645	1.7135	1.853	1.4905
Ptprc	0.8175	4.164	1.46	3.549
Pycard	2.125	10.283	4.1525	8.6085
Rac1	1.3535	1.025	1.6115	0.996
Rbm4	1.063	0.7105	1.0005	0.7815
Rcan1	1.114	4.228	1.5625	3.7745
Rela	0.918	0.5615	0.89	0.618
Rhoa	1.467	1.2145	1.6165	1.274
Rhoa	1.1975	0.935	1.3145	1.064
Rhoa	1.325	1.1075	1.6025	1.12
Rplp2	5.3625	4.5895	5.2165	5.6905
Ryr1	1.405	10.175	0.698	5.8265
S100a8	2.0645	17.282	9.9585	24.7265
S100a9	1.0965	16.975	12.165	27.555
S1pr1	1.52	1.445	2.993	1.3735
S1pr3	1.543	1.574	2.632	2.87
Saa1	1.3865	14.0185	2.067	17.0795
Saa3	1.013	9.131	0.7665	22.794
Scube1	0.774	27.0215	6.245	12.009
Sdcbp	1.246	1.883	1.4495	1.643
Sectm1b	1.1935	15.818	0	9.638
Selp	1.5165	7.5965	7.407	6.462
Serpina1a	1.2145	0.2955	1.292	0.6725
Serpina3c	0.7325	0.4755	0.2465	0.359
Serpinf2	1.259	0.7745	1.7165	1.123
Serping1	1.334	0.712	1.1995	0.8855
Sftpa1	0.669	0.14	0.3795	0.068

Sh2b2	0.824	0.5985	0.839	0.4845
Siglec1	1.1885	4.1765	1.4135	2.8905
Sod1	1.2955	0.5405	1.3745	0.7015
Sod1	1.311	0.523	1.3455	0.7225
Spp1	1.2185	2.771	1.5955	2.6185
Spred1	1.3655	0.9465	1.325	0.908
Spred2	1.27	0.662	0.9215	0.501
Stat3	1.382	1.116	1.499	1.094
Stat4	0.795	2.8555	1.1775	5.364
Stat5b	1.3045	0.8555	1.185	0.8605
Tfrc	1.234	1.8945	1.5535	1.9465
Tgfb1	1.0965	1.7255	1.064	1.119
Tgfb2	4.065	6.318	4.131	9.3265
Tgm2	1.162	1.983	1.475	1.6695
Thpo	1.44	1.1995	1.9725	1.137
Tirap	1.428	0.89	1.5275	1.076
Tlr12	1.965	5.573	2.0095	4.037
Tlr13	1.3725	9.6105	1.3405	7.429
Tlr1	0.769	22.8365	3.2655	13.1215
Tlr2	1.5775	9.1135	1.1605	7.0135
Tlr3	1.44	2.894	2.3215	2.9775
Tlr4	1.1515	4.23	1.4895	3.212
Tlr5	1.4235	0.6995	2.033	0.409
Tlr6	0.956	2.467	1.1865	2.288
Tlr7	1.259	8.4335	1.499	9.1375
Tlr9	1.6695	15.958	4.729	15.551
Tnc	0.5	48.3045	5.728	41.762
Tnf	0.7195	8.0915	0.421	1.908
Tnfaip3	2.2265	11.7095	2.1075	9.49
Tnfrsf11b	0.8935	0.6985	1.03	1.071
Tnfrsf14	1.112	0.898	1.2545	0.9295
Tnfrsf19	1.145	1.3515	1.9215	1.8705
Tnfrsf1b	1.175	1.011	0.746	0.9965
Tnfsf10	0.9815	2.9215	1.239	2.0975
Tnfsf13b	0.5	2.655	1.843	1.7345
Tnfsf14	3.0005	3.9325	0.7685	4.603
Tollip	1.3555	0.694	1.55	0.831
Tpst1	1.1225	0.473	1.324	0.5795
Trem1	1.3645	10.4495	1.9255	13.3485
Trf	1.2625	1.1025	1.3535	1.066
Trpv1	0.894	0	0.8565	0
Tslp	0.831	1.5985	0	1.626

Txlna	1.588	1.1175	1.89	1.184
Ubc	0.9965	0.2345	0.5265	0.3895
Unc13d	1.3995	1.724	0.398	0.608
Vegfa	1.123	0.4175	1.201	0.495
Vegfb	0.9885	0.541	0.8655	0.5135
Vegfc	1.017	1.1665	0.946	1.3435
Xcl1	0.9195	3.675	2.9925	3.995
Xcr1	1.1445	8.1825	1.7825	4.763
Ywhaz	1.3135	1.3365	1.8465	1.909
Zfp36	1.516	0.528	1.696	0.647

Supplementary Figures

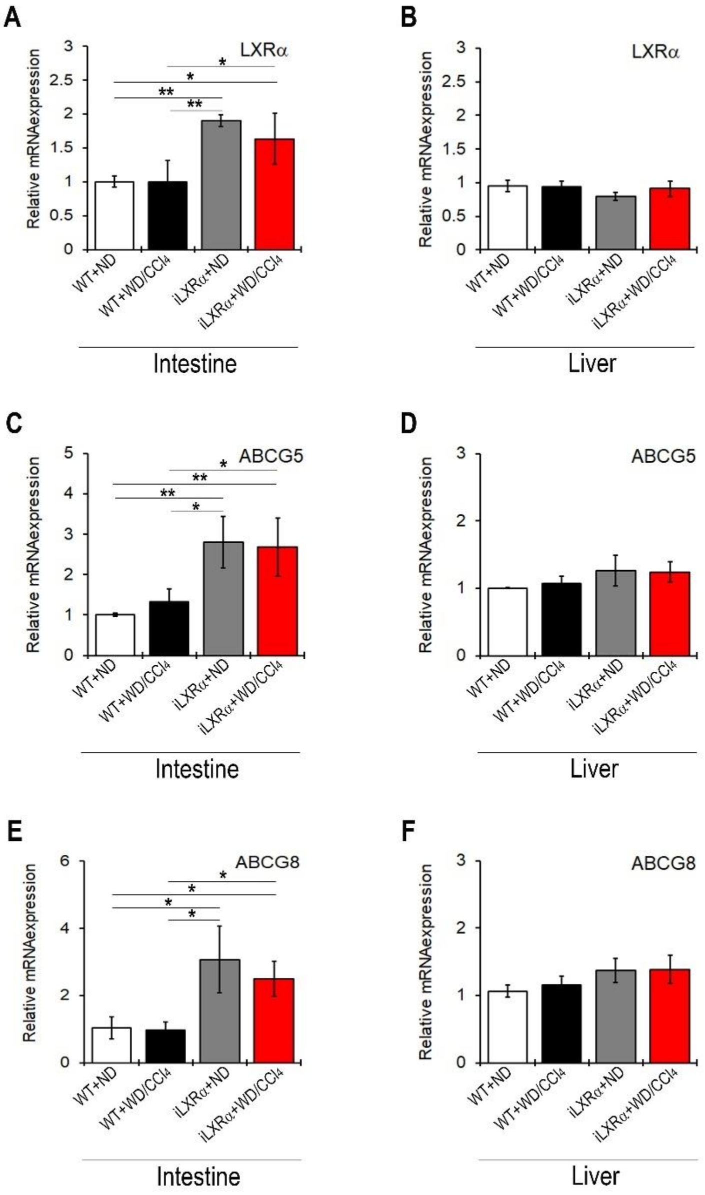

Figure S1. Effect of constitutive intestinal activation of LXRα in WD/CCl$_4$ treated mice on intestinal and hepatic control of cholesterol absorption. The histograms show (**A**) intestinal and (**B**) hepatic mRNA expression of LXRα; (**C**) intestinal and (**D**) hepatic mRNA expression of ABCG5; (**E**) intestinal and (**F**) hepatic mRNA expression of ABCG8 in WT and iLXRα mice treated with ND and WD/CCl$_4$. Gene expression is reported as relative expression by QRT-PCR normalized to GAPDH transcript levels. Data are expressed as the mean ± SD of at least n = 5 animals per group. *p<0.05 and ** p < 0.01 vs. WD/CCl4 and iLXRα.

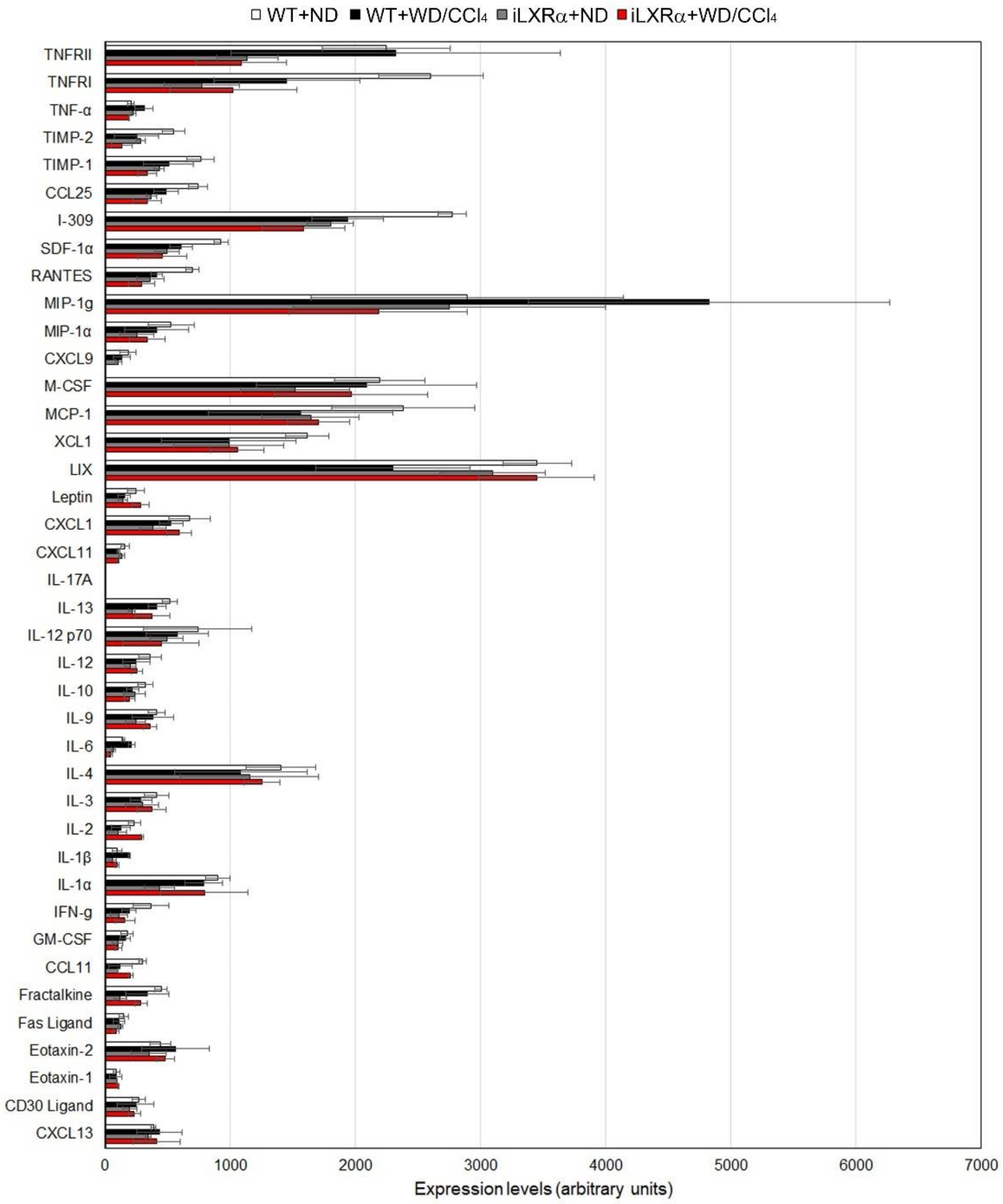

Figure S2. Effect of constitutive intestinal activation of LXR*α* in WD/CCl4 treated mice on circulating cytokines and chemokines. The bar graph shows semiquantitative data of serum cytokine/chemokine expression in WT and iLXR*α* mice treated with ND and WD/CCl4, assessed by a commercial Mouse Inflammation antibody array for 40 targets. Data are expressed as the mean ± SD of at least n = 4 animals per group.

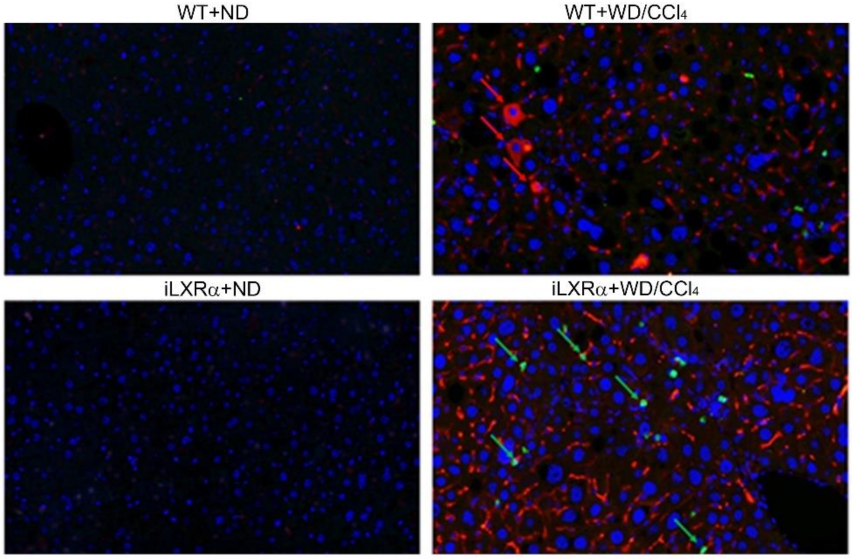

Figure S3. Effect of constitutive intestinal activation of LXRα in WD/CCl4 treated mice on hepatic expression of macrophage markers. Representative confocal imaging by IF of pan-macrophage marker CD68 (red staining and arrows) and M2 macrophage marker CD206 (green staining and arrows) in liver tissue of wild type and iLXRα mice treated with ND and WD/CCl4. Hoechst was used for nuclear staining (blue). Magnification 60x.

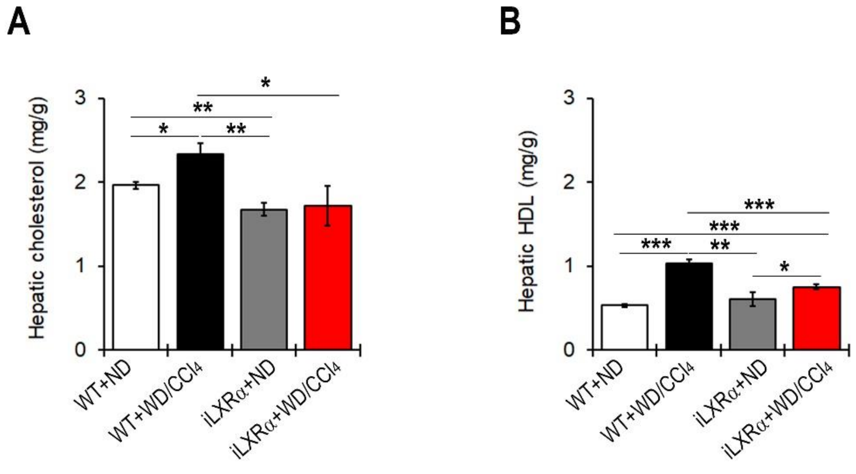

Figure S4. Effect of constitutive intestinal activation of LXRα in WD/CCl4 treated mice on hepatic cholesterol and HDL content. The bar graphs show the quantification of (**A**) cholesterol levels and (**B**) HDL levels in liver extracts from WT and iLXRα mice treated with ND and WD/CCl4. Data are expressed as the mean ± SD of at least n = 5 animals per group. *p<0.05, ** p < 0.01 and *** p < 0.001 vs. WD/CCl4 and iLXRα.

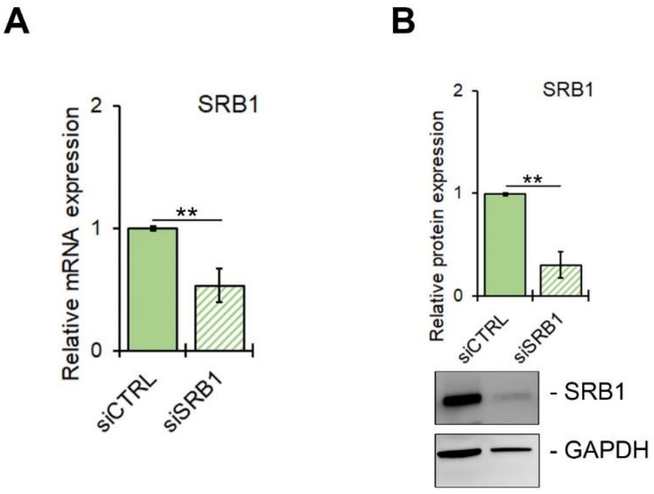

Figure S5. The effect of SRB1 silencing on the expression of SRB1 transcript and protein in HepG2 cells after 24 hrs from silencing. The bar graphs show (**A**) mRNA expression of SRB1; and (**B**) quantitative densitometry and representative immunoblot of SRB1. Gene expression is reported as a relative expression by QRT-PCR normalized to GAPDH transcript levels. Protein quantification was performed by Western blotting and normalization against GAPDH. Data are expressed as the mean ± SD of at least n = 3 independent experiments. ** p < 0.01.

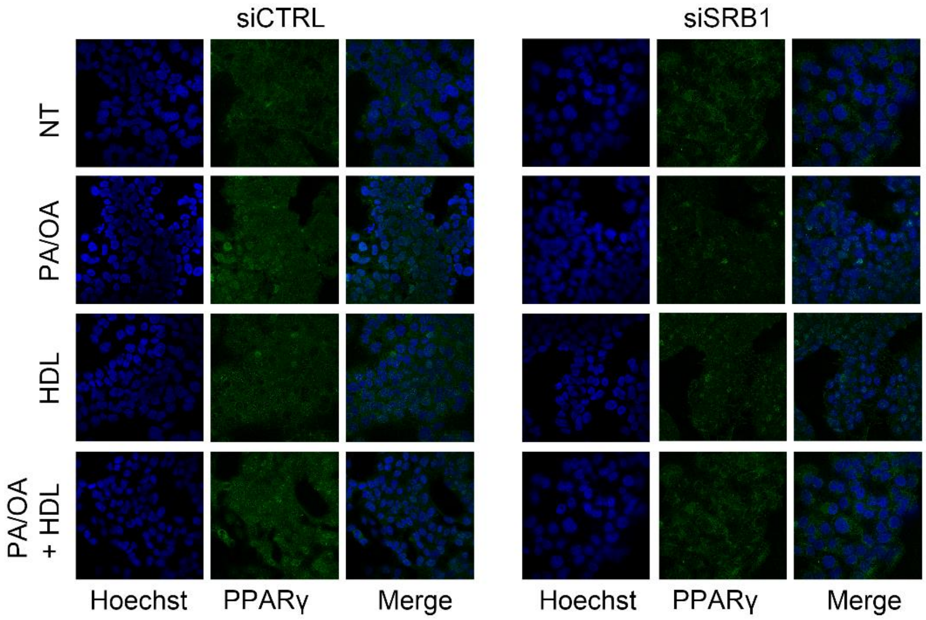

Figure S6A. Single staining in the image of panel E in Figure 6. The image includes the representative uncropped confocal imaging by IF (Magnification 60x) of the PPARγ (green), nuclei-Hoechst (blue), and merge in siCTRL and siSRB1 HepG2 cells treated with FFAs (PA/OA) alone or in combination with pre-exposure to HDL and collected after 24 hrs;

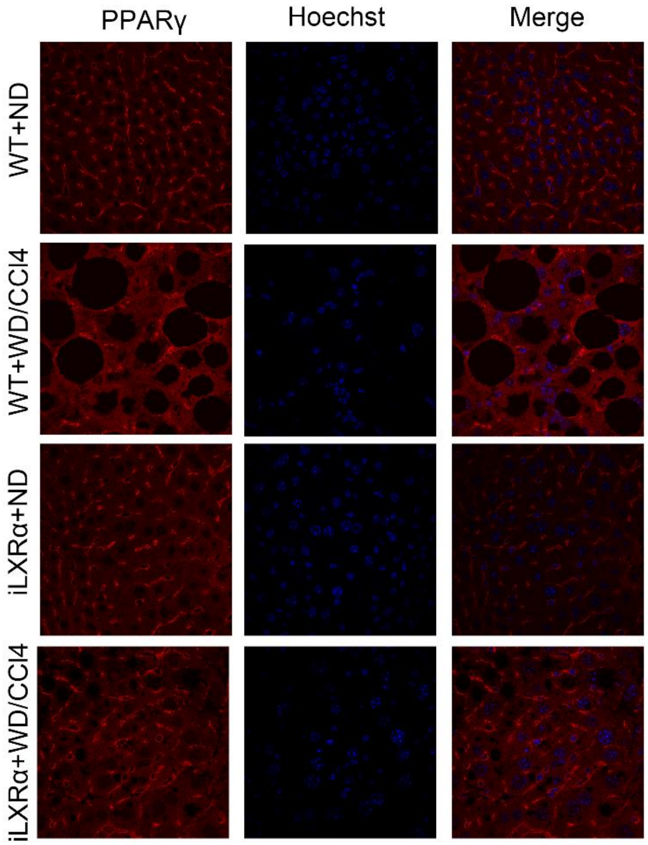

Figure S6B. Uncropped image of panel F in Figure 6.

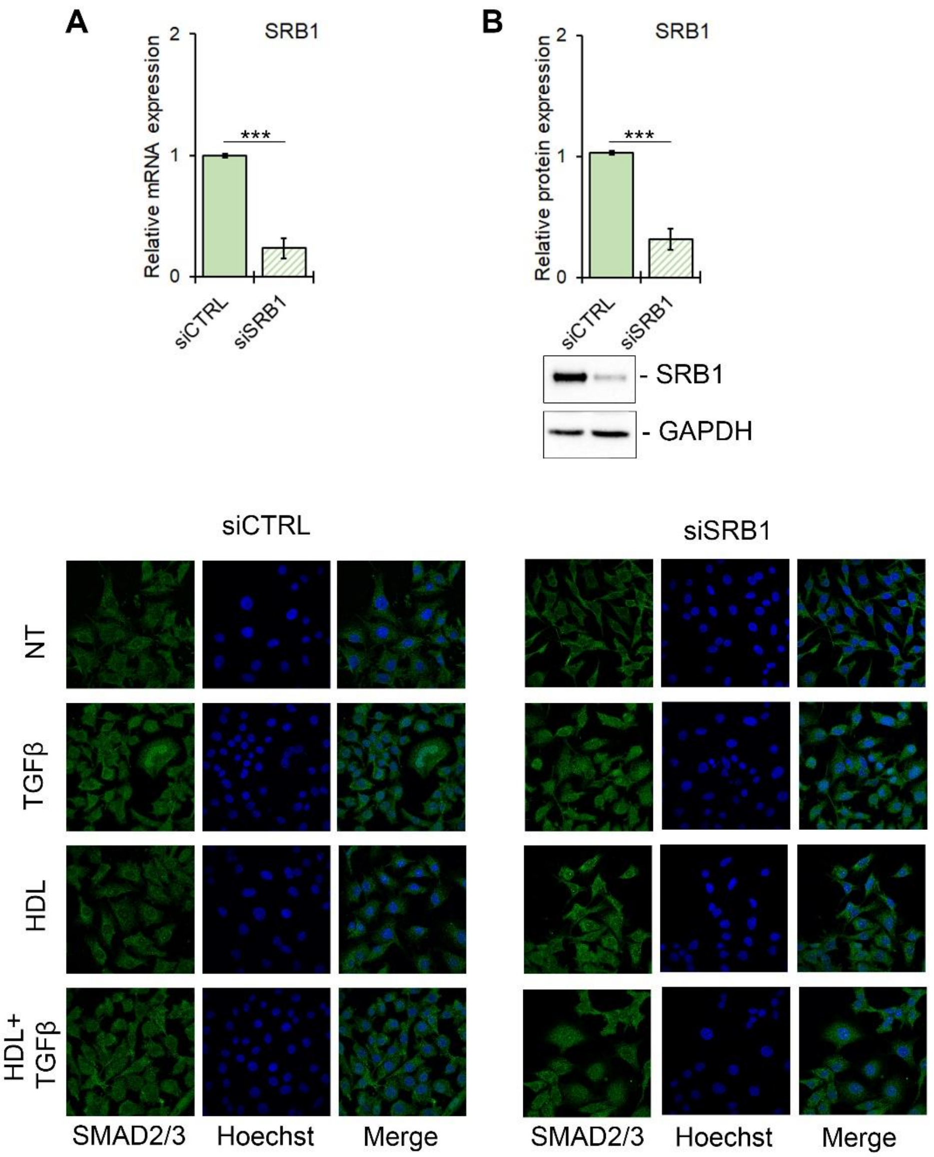

Figure S7. The effect of SRB1 silencing on the expression of SRB1 transcript and protein in LX2 cells after 24 hrs from silencing. The bar graph shows (**A**) mRNA expression of SRB1; and (**B**) quantitative densitometry and representative immunoblot of SRB1. Gene expression is reported as a relative expression by QRT-PCR vs siCTRL normalized to GAPDH transcript levels. Protein quantification was performed by Western blotting and normalization against GAPDH. Data are expressed as the mean ± SD of at least n = 3 independent experiments. *** $p < 0.001$; (**C**) Uncropped image of panel E in Figure 7. The image includes the representative uncropped confocal imaging by IF (Magnification 60x) of SMAD2/3 (green), nuclei-Hoechst (blue), and merge in siCTRL and siSRB1 LX-2 cells treated with TGFβ alone or in combination with pre-exposure to HDL and collected after 24 hrs.

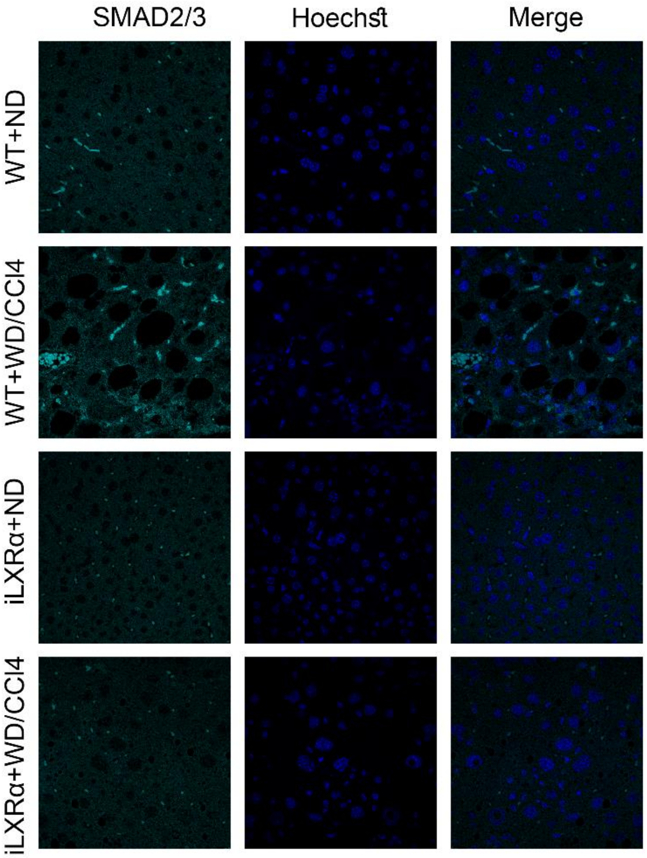

Figure S8. Uncropped image of panel F in Figure 7.
